# Artificial intelligence applications in refractive error management: A systematic review and meta-analysis

**DOI:** 10.1371/journal.pdig.0000904

**Published:** 2025-09-25

**Authors:** Josephine Ampong, Sylvia Agyekum, Werner Eisenbarth, Albert Kwadjo Amoah Andoh, Isaiah Osei Duah Junior, Gabriel Amankwah, Gabriel Kwaku Agbeshie, Eldrick Adu Acquah, Clement Afari, Emmanuel Assan, Saphiel Osei Poku, Karen Ama Sam, Josephine Ampomah Boateng, Kwadwo Owusu Akuffo

**Affiliations:** 1 Department of Optometry and Visual Science, College of Science, Kwame Nkrumah University of Science and Technology, Kumasi, Ghana; 2 Department of Applied Sciences and Mechatronics, Hochschule München University of Applied Sciences, Munich, Germany; 3 Department of Chemistry, College of Science, Kwame Nkrumah University of Science and Technology, Kumasi, Ghana; Liverpool John Moores University - City Campus: Liverpool John Moores University, UNITED KINGDOM OF GREAT BRITAIN AND NORTHERN IRELAND

## Abstract

Artificial intelligence (AI) has transformed healthcare, and is becoming increasingly useful in eye care. We conducted a systematic review and meta-analysis of the use of AI in the diagnosis, detection, prediction, progression, and treatment of refractive errors (REs). The study adhered to the PRISMA checklist to ensure transparent reporting. The following databases were searched from inception to January 2025, with an English language restriction: PubMed, Web of Science, Embase, Scopus, Cochrane Library and Google Scholar. Two independent reviewers performed study screening, data extraction, and quality assessment, with a third author resolving discrepancies. All original studies on the use of AI techniques in RE were identified and the effectiveness of these techniques was compared. A critical appraisal was conducted using the QUADAS-2 risk-of-bias tool. A meta-analysis was performed using R software (version 4.5.0). Of 6,288 records retrieved, 45 met eligibility for systematic review, with 19 included in meta-analysis. Among these 45 studies, 55.5% (25/45) applied deep learning (DL) approaches, while 44.4% (20/45) employed machine learning (ML) techniques. The pooled sensitivity, specificity, diagnostic odds ratio (DOR), and summary of receiver operating characteristic (SROC) for detection and/or diagnosis studies were 0.94 (95%CI, 0.90-0.97), 0.96 (95%CI, 0.92-0.98), 382.56 (95% CI 111.91 -1307.77) and 0.98 (95%CI, 0.91-0.97), respectively. For prediction of REs, the pooled sensitivity, specificity, DOR, and SROC were 0.87 (95%CI, 0.73-0.94), 0.96 (95%CI, 0.90-0.980), 159.94 (95% CI, 40.17-636.85) and 0.96 (95%CI, 0.85-0.95), respectively. Among studies focused on progression, performance metrics ranged from AUC = 0.845-0.99, R² = 0.613-0.964, and MAE = 0.119D-0.49D. In treatment studies, performance varied more widely, with AUC values between 0.60–0.94 and MAE from 0.17D-0.54D. Collectively, AI technologies, particularly DL and ML, achieved high diagnostic and predictive accuracy in RE management. Future research should focus on developing generalizable models trained on diverse datasets to ensure broad clinical relevance.

## Author summary

Refractive errors, such as nearsightedness, farsightedness, and astigmatism, are common vision problems that affect people of all ages. They can usually be corrected with glasses, contact lenses, or surgery, but early and accurate detection is key to effective treatment. In recent years, AI has become a valuable tool in improving how these conditions are diagnosed, monitored, and managed. We investigate how AI is being used to detect, predict, track progression, and help treat these conditions. We analyzed 45 studies, of which 19 were included in a detailed meta-analysis. Over half of the included studies used DL, a type of AI that mimics the human brain, while the others used ML, an AI approach where computers identify patterns from data. We found that AI tools were very accurate at diagnosing and predicting refractive errors, with high sensitivity (correctly identifying those with the condition) and specificity (correctly identifying those without it). Further, the AI was also useful in tracking changes in vision over time and in helping guide treatment choices, although performance varied more in those areas. Overall, AI shows strong potential to improve how we detect and manage refractive errors. Future work should focus on developing AI systems that work well across different populations and clinical settings.

## Background

Refractive errors (REs) are among the most common vision problems, affecting millions of people worldwide—from infancy to old age [1]. These REs, including myopia, hyperopia, and astigmatism, arise from structural abnormalities in the eye that prevent proper light focus on the retina, resulting in blurred vision [2,3]. According to the World Vision Report [4], over 2.2 billion people are visually impaired or blind, including 101.2 million with moderate to severe visual impairment and 6.8 million cases of blindness due to uncorrected refractive errors (UREs) [5]. If left untreated, UREs can significantly impact individuals and communities by reducing quality of life, limiting education and employment opportunities, and hindering economic productivity [6]. Visual impairments due to UREs are projected to rise [7], highlighting the urgent need for enhanced screening and management strategies for early detection [8].

Traditional methods for diagnosing and managing REs rely on several eye tests such as visual acuity testing [9], objective refraction [10] and subjective refraction [11]. Objective techniques such as autorefraction and retinoscopy offer quicker, more standardized alternatives. However, autorefraction, especially without cycloplegia, is prone to overestimating myopia or underestimating hyperopia due to accommodation, while retinoscopy - though accurate, requires technically skilled personnel, which may not be feasible during high-volume screening. Subjective refraction requires patient input, which may be unreliable - particularly in pediatric populations [12,13], and individuals with intellectual disabilities [14]. Additionally, it can be time-consuming, especially in large-scale screenings [12,15]. Effective intervention requires an early detection, monitoring of REs to avert the associated pathological challenges [16]. However, the growing burden of UREs may overwhelm existing eye healthcare systems which calls for exploitation of optimally smart strategies [17].

Artificial intelligence (AI) enables computer systems to perform tasks requiring human intelligence, such as problem-solving, reasoning, learning, and decision-making [18,19]. Its primary goal is to enhance computational power and automate tasks with minimal human input, boosting productivity and efficiency [20]. AI has become a transformative technology with broad impacts across industries, including ophthalmology [21,22]. It has demonstrated strong effectiveness in diagnosing various eye conditions, such as age-related macular degeneration [23,24], cataract [25,26], diabetic retinopathy [27,28], and glaucoma [29].

Artificial intelligence encompasses technologies such as ML and DL [30]. Machine learning algorithms excel at analyzing complex, non-linear relationships between predictors and outcomes, improving predictive accuracy [31]. Similarly, DL algorithms are particularly effective at interpreting intricate ocular images, detecting subtle patterns that distinguish healthy from abnormal eyes [30]. In the context of REs, ML and DL have shown strong performance in detection [34], diagnosis [32,33], prediction [34], monitoring progression [35], and treatment [36] utilizing large datasets of images and clinical data [37,38]. These successes demonstrate AI’s ability to extract critical features from complex ophthalmic data, enhancing accuracy, efficiency, and personalized vision care [39–41]. This systematic review and meta-analysis aims to provide comprehensive evidence from the current literature on AI applications in REs, with a focus on its roles in detection, diagnosis, prediction, monitoring, and treatment.

## Methods

**Fig 1:**
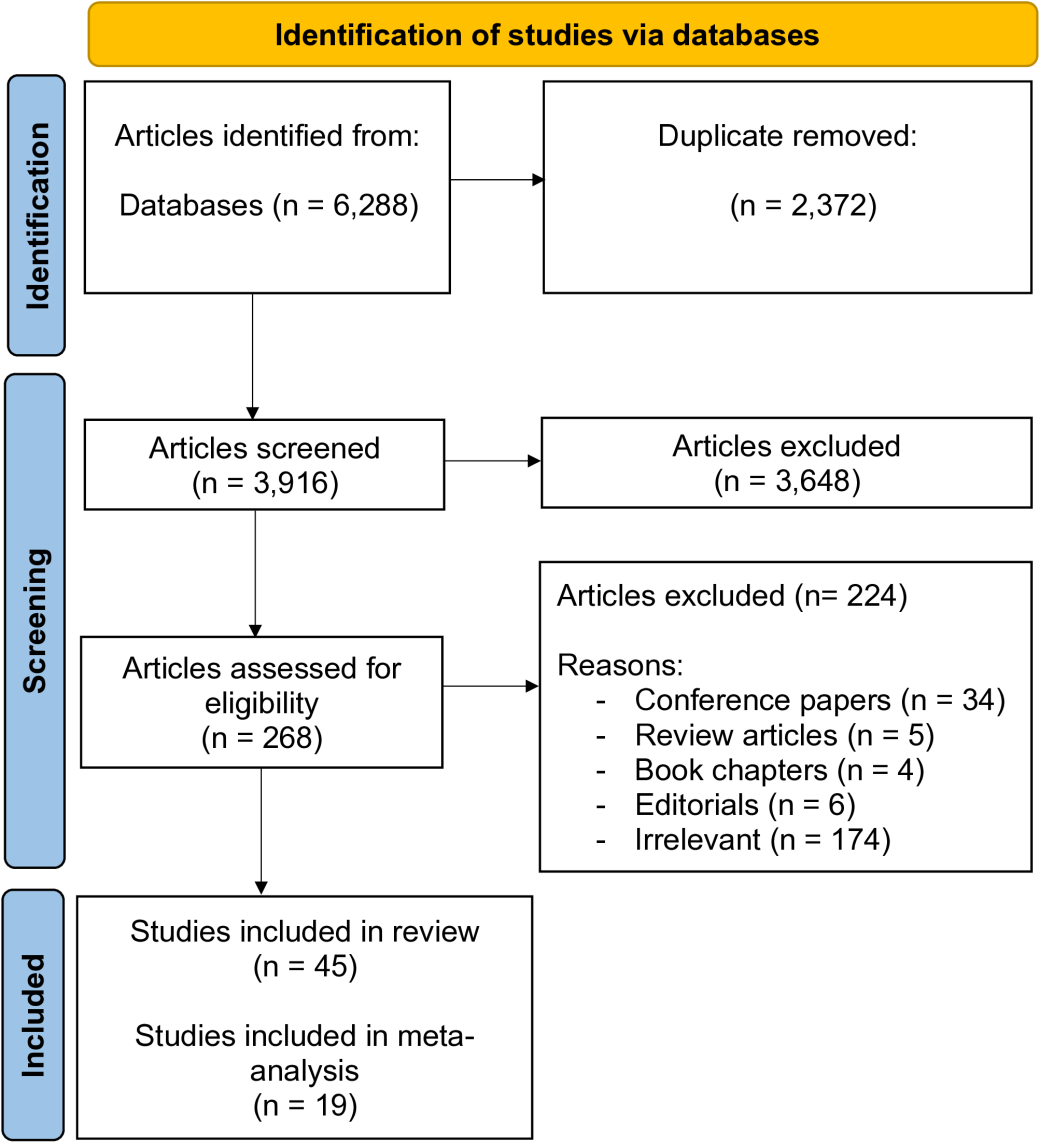
PRISMA flow chart showing the study retrieval and study selection.

This protocol was registered with PROSPERO (CRD42024512157). The systematic review and meta-analysis followed Preferred Reporting Items for Systematic Review and Meta-analysis (PRISMA) checklist for transparency of reporting (S1 PRISMA Checklist) [42] and adhered to methods outlined in the Cochrane Handbook for robustness and reproducibility [43]. The study selection process and database screening were systematically documented using the PRISMA flow diagram (**Fig 1**) [44].

### Eligibility criteria

We included original, full-text articles published in peer-reviewed English-language journals that applied AI techniques (e.g., DL and ML), to ophthalmic imaging and/or clinical data to address REs. Studies involving non-human subjects, case reports, case series, reviews, commentaries, editorials, and opinion pieces were excluded.

### Search strategy

A literature search was performed in PubMed, Web of Science, Embase, Scopus, Cochrane library and Google Scholar for peer-reviewed articles published up to January 2025. The search combined terms related to REs (e.g., myopia, hyperopia, astigmatism), AI (e.g., machine learning, deep learning), and management (e.g., diagnosis, prediction, treatment). Detailed search strings are provided in S2 Text.

### Study selection

All publications identified from the databases were imported into Covidence software [45] for processing, including duplicate removal, title and abstract screening, and full-text review. Two authors (JA and KAS) independently screened titles and abstracts based on predefined inclusion and exclusion criteria. Studies passing this stage underwent full-text review for final inclusion. Any disagreements were resolved through discussion between the reviewers. Additionally, reference lists of potentially relevant studies were examined to identify any missed articles.

### Data extraction

The primary outcome was to identify AI techniques used in RE diagnosis, detection, prediction, progression, and treatment, and to evaluate their effectiveness in RE management. Two authors (JA and EA) independently extracted data using a Covidence-designed extraction sheet. Extracted data included study characteristics (author, year, country, design, sample size), type of images or data, AI techniques and models used, performance metrics, and main outcomes. Discrepancies were resolved through discussion, with a third reviewer (SA) consulted for complex issues.

### Quality Assessment

Risk of bias was assessed using the QUADAS-2 tool, which evaluates four domains: patient selection, index test, reference standard, and flow and timing [46]. Each domain was rated as high, low, or unclear risk of bias. The first three domains were also assessed for applicability concerns using the same rating scale. Signaling questions guided the evaluations and two authors (JA and SOP) independently performed the assessments, resolving disagreements through discussion and consensus.

### Data analysis

Quality assessment was performed using RevMan 5.3 (Cochrane Collaboration, Denmark), and meta-analysis was conducted in R (version 4.5.0). Studies were eligible for meta-analysis if they provided sufficient data to construct 2 × 2 diagnostic tables, or reported sensitivity and specificity values. For studies evaluating multiple algorithms, results from the highest-performing algorithm per dataset were selected. When 2 × 2 diagnostic tables were unavailable, calculations were derived from sensitivity, specificity, precision, or F1 scores along with case numbers. Meta-analysis grouped studies into detection and/or diagnosis and prediction categories, using random-effects models to synthesize data. Pooled analyses assessed diagnostic performance indicators such as sensitivity, specificity, DOR, and SROC. Study heterogeneity and threshold effects were also evaluated.

Heterogeneity among studies was assessed using the I² statistic, with values ≥50% indicating substantial heterogeneity [47]. Threshold effects were evaluated via Spearman’s correlation between logit-transformed sensitivity and 1-specificity, with p < 0.05 deemed statistically significant. Subgroup analyses were planned based on input modalities and AI technique covariates. Publication bias was assessed using Egger’s funnel plots and asymmetry tests. Progression and treatment studies were synthesized narratively.

### Dealing missing data

Only records with sufficient information for primary outcomes were included. Missing data were not imputed.

### Ethics and dissemination

As this study used secondary data, ethical approval was not required. Findings will be shared with stakeholders, presented at scientific conferences, published in a peer-reviewed journal, and disseminated via public social media platforms.

## Results

The literature selection process is summarized in a PRISMA flow diagram (**Fig 1**). The initial search identified 6,288 articles. After removing 2,372 duplicates, 3,916 titles and abstracts were screened, excluding 3,648 articles. This left 268 full-text articles for eligibility assessment, of which 224 were excluded (conference papers, reviews, book chapters, editorials, or irrelevant). Ultimately, 45 studies were included in the review, with 19 eligible for meta-analysis.

### Study characteristics

The characteristics of the 45 included studies are summarized in **Table 1**. These studies were published between 2013 and 2024. Most were conducted in Asia, with 26 (57.8%) in China and 8 (17.8%) in Korea/South Korea. The remainder originated from Singapore, India, New Zealand, the UK, and Croatia. Study designs were primarily retrospective (n = 22 studies), followed by cross-sectional (n = 3 studies), longitudinal (n = 4 studies), and one each of prospective [48], longitudinal cross-sectional [49], and retrospective clinical trial [50]. Thirteen studies did not specify their design, and regarding validation, 31 studies used internal validation only, while 14 applied both internal and external validation.

**Table 1:** Characteristics of included studies.

Author, Year	Study Country	Study Design	Total sample size		Type of Images/Data Used	AI Technique	Model used	Validation type	Model Aim
			Patients	Images/Data					
Li *et al*. 2023 [51]	China	Retrospective	497	N/A	Corneal topography maps	ML	Bagging Tree Gaussian Process SVM Decision Tree	Internal	Estimating the original corneal curvature after ortho-k
Varosanec *et al*. 2024 [52]	Croatia	Longitudinal	895	10,170	Clinical data	DL	RNN: Extended gate time-aware long short-term memory	Internal	Predicting future spherical equivalent
Li *et al*. 2024a [53]	China	Retrospective	227,543	612,530	Clinical data	ML	Multivariate linear regression Logistic regression	Internal External	Predicting the progression of myopia and progression to high myopia
Lu *et al*. 2021a [54]	China	Retrospective	28,913	32,010	Fundus images	DL	CNN: ResNet-18 Faster R-CNN	Internal External	Identifying non-pathologic myopia and pathologic myopia
Jiang *et al*. 2023 [55]	China	Retrospective	1678	2767	Clinical data	ML	Support vector regression Random Forest XGBoost LASSO	Internal	Predicting postoperative refraction errors
Pathan *et al*. 2020 [56]	N/A	N/A	N/A	400	Fundus images	ML	Multilayer perceptron AdaBoost	Internal External	Detecting pathological myopia and non-pathological myopia
Rauf *et al.* 2021 [57]	N/A	N/A	N/A	400	Fundus images	DL	CNN	Internal External	Detecting pathological myopia
Zhang *et al*. 2013 [58]	China	Cross-sectional	2,258	N/A	Fundus images Clinical data Genotyping data	ML	SVM	Internal	Detecting pathological myopia
Peng *et al*. 2024 [59]	China	N/A	2492	N/A	Fundus images	DL	Attention-based Patch Residual Shrinkage network	Internal	Diagnosing paediatric high myopia
Yang *et al*. 2019 [60]	China	Retrospective	N/A	2350	Ocular appearance images	DL	DCNN: VGG-Face	Internal	Identifying the presence of myopia
Linde *et al*. 2023 [61]	New Zealand India	Retrospective	512	N/A	Pupillary red reflex images	DL	CNN: Inception-V3 EfficientNet	Internal	Estimating refractive error
Xu *et al*. 2022 [62]	China	N/A	N/A	3103	Photorefraction images	DL	CNN + RNN	Internal	Predicting refractive error
Varadarajan *et al*. 2018 [63]	United Kingdom	N/A	64755	N/A	Fundus images	DL	ResNet	Internal	Predicting refractive error
Yoo *et al*. 2021 [64]	South Korea	Retrospective	468	936	OCT images	DL	ResNet50 InceptionV3 VGG16	Internal External	Estimating uncorrected refractive error
Park *et al*. 2022 [65]	Korea	Retrospective	367	36700	OCT images	DL	CNN: ResNet18 ResNext50 EfficientNetB0 EfficientNetB4	Internal	Distinguishing between pathological myopia group and normal group
Choi *et al*. 2021 [66]	Korea	Retrospective	436	1,200	OCT images	DL	CNN: VGG 16 ResNet 50 Inception V3	Internal	Distinguishing high myopia from normal and other retinal diseases
Foo *et al*. 2023 [67]	Singapore	Retrospective	965	7456	Fundus images Clinical data	DL	DNN: DenseNet-121 Random Forest	Internal External	Predicting the development of high myopia
Chun *et al*. 2020 [68]	Korea	N/A	164	305	Photorefraction images	DL	CNN: ResNet-18	Internal	Predicting the range of refractive error
Zhang *et al*. 2024 [69]	China	Retrospective	369	1346	Corneal topography maps	DL	DNN: Segformer Architecture Network	Internal	Determining the Treatment Zone and Peripheral Steepened Zone following ortho-K
Jain *et al*. 2024 [70]	South Korea India	Cross-sectional	1331	2662	OCT images	DL	CNN: ResNet50	Internal	Predicting uncorrected refractive error
Yang *et al*. 2024 [71]	China	Retrospective	266	449	Clinical data	DL	DNN: Dense	Internal	Predicting lens prescription parameters in ortho-K
Koo *et al*. 2024 [72]	Korea	Retrospective	297	547	Clinical data	ML	Decision tree CatBoost Extra Trees XGBoost Random Forest Least Angle regression Ridge LASSO	Internal	Selecting ortho-K lens parameters
Lu *et al*. 2021b [73]	China	Retrospective	13869	17330	Fundus images	DL	CNN: DenseNet201 ResNet50 VGG16 Xception	Internal External	Detecting pathological myopia
Peng *et al*. 2023 [74]	China	Prospective	2538	N/A	Clinical data	ML	Random Forest SVM Gradient Boosting Decision Tree CatBoost	Internal	Predicting the onset of myopia
Ren *et al*. 2023 [75]	China	Retrospective	N/A	1156	Fundus images	DL	ResNet18 Faster R-CNN	Internal	Diagnosing pathological myopia
Yoo *et al*. 2020 [76]	Korea	Retrospective	18,480	N/A	Clinical data	ML	XGBoost SVM Random forest ANN	Internal External	Selecting best laser refractive surgery option
Fan *et al*. 2022 [77]	China	Retrospective	1,271	N/A	Clinical data	ML	Linear regression SVM Bagging decision trees Gaussian processes	Internal	Estimating alignment curve curvature in ortho-K lens fitting
Fang *et al*. 2023 [50]	China	Retrospective clinical trial	91	N/A	Clinical data	ML	LASSO regression	Internal	Predicting the treatment effect of ortho-k
Xu *et al*. 2023 [78]	China	Retrospective	1,302	N/A	Clinical data	ML	SVM Gaussian process regulator Decision tree Random Forest	Internal	Predicting ortho-K lens parameters and axial length progression
Ying *et al*. 2024 [31]	China	Cross-sectional	3,414	6,827	Clinical data	ML	SVM Random Forest XGBoost MLP-NN Linear regression LASSO regression	Internal External	Predicting cycloplegic SER and myopia status
Kim *et al*. 2021 [79]	South Korea	Retrospective	1839	N/A	Fundus images Clinical data	ML	SVM Decision Tree Random Forest K-nearest neighbors Naïve Bayes classifiers	Internal	Predicting pathological myopia
Huang *et al*. 2023 [80]	China	Retrospective	37586	75172	Clinical data	DL	RNN: T-LSTM Standard LSTM Random Forest Linear Regression	Internal	Predicting myopia
Zhao *et al*. 2024a [81]	China	N/A	N/A	7,114	Fundus images	DL	CNN: ResNet-101	Internal	Classifying pathological myopia
Hemelings *et al*. 2021 [82]	N/A	N/A	N/A	1200	Fundus images	DL	ResNet-18 U-Net++	Internal External	Detecting and classifying pathological myopia
Yang *et al*. 2020 [83]	China	N/A	3112	N/A	Clinical data	ML	SVM	Internal	Studying influence of related factors to predict myopia
Barraza-Bernal *et al*. 2023 [84]	China	Cross-sectional	12780	N/A	Clinical data	ML	Gaussian process regression Support vector regression Support vector machine (SVM)	Internal	Predicting refractive error its development over time
Yang *et al*. 2022 [85]	China	N/A	987	987	Fundus images	DL	ResNet-50 Inception-v3 Inception-ResNet-v2	Internal External	Predicting refractive error in myopic patients
Yuan *et al*. 2023 [86]	China	N/A	N/A	14,028	Clinical data	ML	BP neural network	Internal	Predicting cutting formula of SMILE
Li *et al*. 2024b [87]	China	Longitudinal	12,766	12,766	Clinical data	ML	XGBoost K Neighbors Decision tree Logistic regression Gaussian NB	Internal	Predict myopia progression and the risk of developing high myopia
Zhu *et al*. 2023 [88]	China	Retrospective	N/A	179	Clinical data	ML	Orthogonal matching pursuit Random Forest Kernel ridge regression K-nearest neighbor regression Extra tree regression Multilayer perceptron	Internal	Predict the changes in SER and AL
Tan *et al*. 2021 [89]	Singapore	Retrospective	112,110	225,671	Fundus images	DL	CNN: ResNet-101	Internal External	Detecting high myopia
Ali and Raut. 2024 [90]	N/A	N/A	N/A	400	Fundus images	DL	CNN: Spatial attention network Squeeze- excitation network	Internal External	Detecting pathological myopia
Zhao *et al*. 2024 b [91]	China	Longitudinal	88,250	408,255	Clinical data	ML	Random forest XGBoost	Internal	Predict SE and development of myopia and high myopia
Lin *et al*. 2018 [92]	China	Longitudinal	129242	687063	Clinical data	ML	Random forest	Internal External	Predict the onset of high myopia, at specific future time points
Tang *et al*. 2021 [93]	China	N/A	2044	6328	Corneal topography maps	DL	DNN + CNN	Internal	Identify treatment zone boundary and treatment zone center

DL Deep learning; ML Machine learning; CNN Convolutional neural network; RNN Residual neural network; DNN Deep neural network; DCNN Deep convolutional neural network; SMILE Small incision lenticule extraction; SVM Support vector machine; Ortho-K Orthokeratology

### AI applications

**Fig 2:**
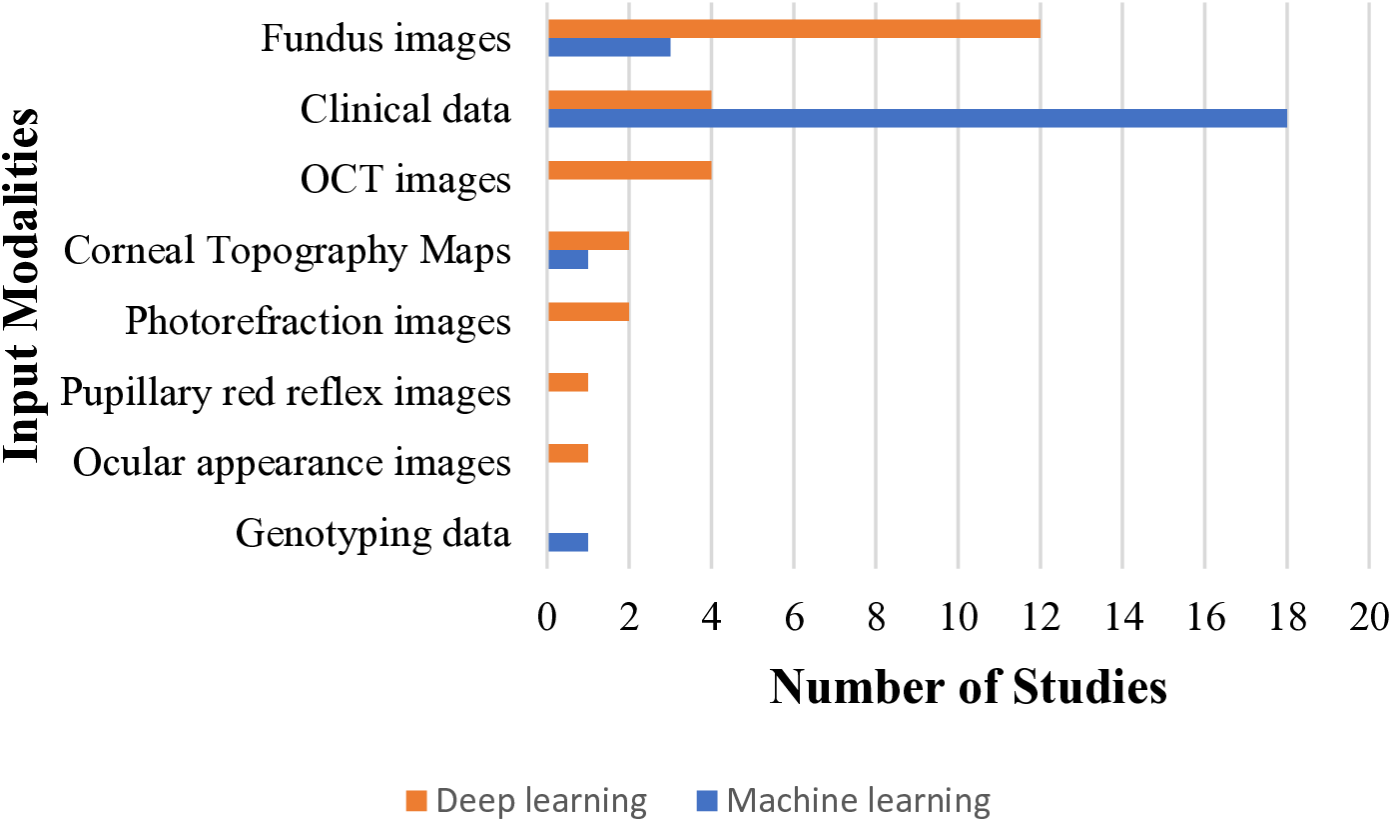
Types of AI techniques and input modalities used in the included studies.

AI applications in RE management included detection and/or diagnosis (n = 14 studies), prediction (n = 15), progression monitoring (n = 5 studies), and treatment (n = 11 studies). Deep learning (DL) was the most used technique (n = 25 studies), followed by machine learning (ML) in 20 studies. Common DL architectures included ResNet (n = 13 studies), Inception V3 (n = 4 studies), and VGG Face (n = 4 studies). Among ML models, support vector machine (SVM) (n = 9 studies), random forest (n = 10 studies), and XGBoost (n = 6 studies) were prevalent. AI methods analyzed retinal fundus images (n = 15 studies), OCT images (n = 4 studies), clinical data (n = 22 studies), eccentric photorefraction images (n = 4 studies), corneal topography maps (n = 2 studies), ocular appearance images (n = 1 study), genotyping data (n = 1 study), or combinations thereof (See **Table 1 and Fig 2**).

### Risk of bias of studies

The included studies were of moderate to high quality. Forty-one studies (91.1%) were rated as low risk of bias across all four QUADAS-2 domains. In patient selection, 4 studies (8.8%) had unclear risk and applicability concerns due to insufficient dataset descriptions. Most studies (91.1%) showed low risk and concerns in the index test domain, with 4 studies (8.8%) marked unclear due to data overlap. All studies had low risk in the reference standard domain and for flow and timing, 3 studies (6.7%) were rated unclear because of poor documentation on dataset assembly (see S3 Table).

### AI in refractive error detection and diagnosis

**Fig 3:**
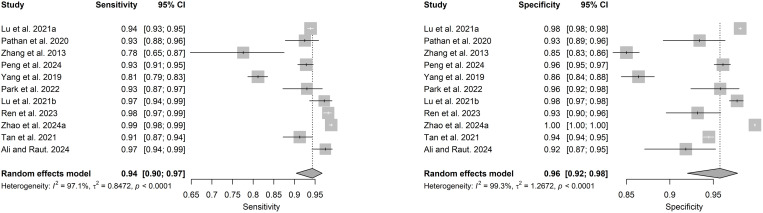
Forest plots for sensitivity (3A) and specificity (3B) for AI in refractive error detection or diagnosis.

**Fig 4:**
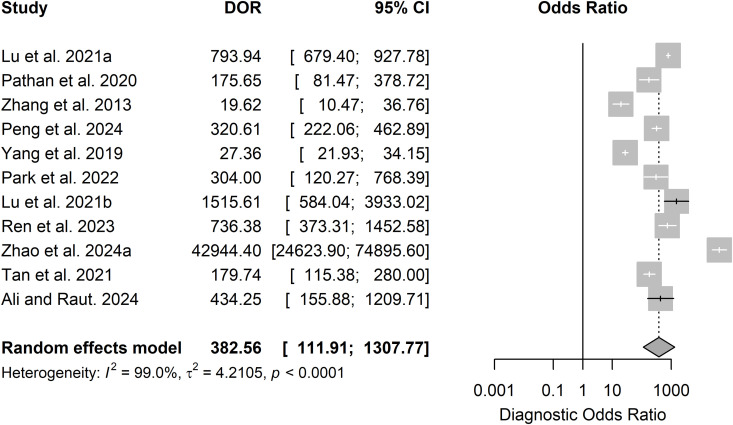
Forest plot for DOR for detection or diagnosis studies.

**Fig 5:**
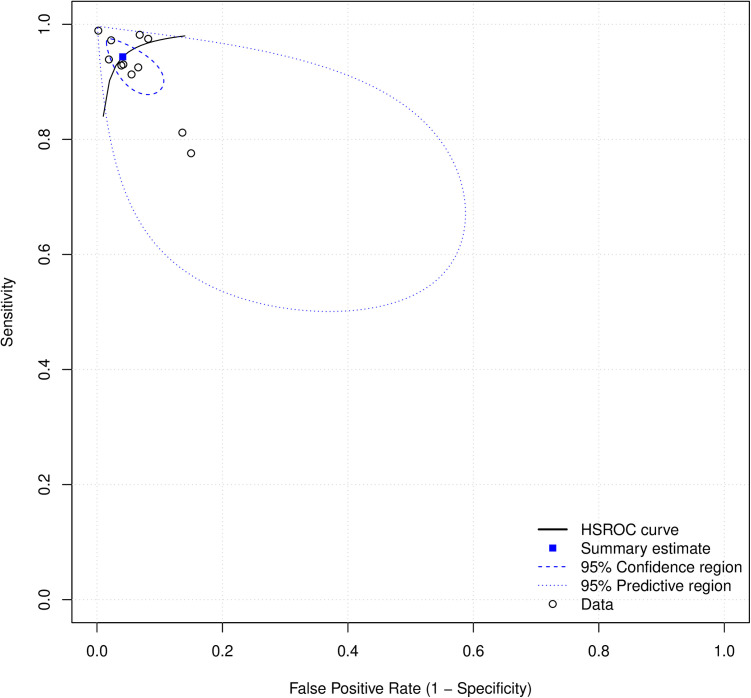
SROC curve for detection or diagnosis studies.

Out of 14 studies on detection and/or diagnosis of refractive errors, 11 were included in the meta-analysis. The pooled sensitivity was 0.94 (95% CI: 0.90–0.97) and specificity 0.96 (95% CI: 0.92–0.98) (see **Fig 3**). The DOR was 382.56 (95% CI: 111.91–1307.77) and the SROC was 0.98 (95% CI: 0.91–0.97) as shown in Figs 4 and 5.

### Threshold analysis for detection and diagnosis studies

Threshold analysis showed no significant correlation (Spearman r = -0.536, p = 0.094), indicating that variability among studies was unlikely due to differing diagnostic thresholds.

### Publication bias for detection and diagnosis studies

Egger’s funnel plot asymmetry test showed no publication bias (P = 0.902), as illustrated in S4 Fig.

### Subgroup analysis by AI technique for detection and diagnosis studies

Nine studies used DL as the AI model backbone, with pooled sensitivity of 0.95 (95% CI: 0.92–0.97), specificity 0.97 (95% CI: 0.93–0.98), and DOR 580.30 (95% CI: 147.92–2276.57). The remaining two studies used ML, showing pooled sensitivity of 0.87 (95% CI: 0.65–0.96), specificity 0.90 (95% CI: 0.78–0.95), and DOR 58.02 (95% CI: 6.77–497.17). A significant difference was found in pooled specificity (p = 0.04) between DL and ML, but no significant differences were observed for sensitivity (p = 0.11) or DOR (p = 0.08) –see S5 Fig.

### Subgroup analysis by input modalities for detection and diagnosis studies

Eight studies used fundus images, while one study each used optical coherence tomography (OCT) images, ocular appearance images, and a combination of fundus images, clinical data, and genotyping data. For fundus images, pooled sensitivity was 0.96 (95% CI: 0.94–0.98), specificity 0.97 (95% CI: 0.95–0.98), and DOR 801.47 (95% CI: 286.63–2241.08). The ocular appearance image study showed sensitivity of 0.81 (95% CI: 0.79–0.83), specificity of 0.86 (95% CI: 0.84–0.88), and DOR of 27.36 (95% CI: 21.93–34.15). The OCT image study reported sensitivity of 0.93 (95% CI: 0.87–0.97), specificity of 0.96 (95% CI: 0.92–0.98), and DOR of 304.00 (95% CI: 120.27–768.39). The combined modality study yielded sensitivity of 70.8 (95% CI: 0.65–0.87), specificity of 0.85 (95% CI: 0.83–0.86), and DOR of 19.62 (95% CI: 10.47–36.76). Significant differences in pooled sensitivity, specificity, and DOR were observed between input modalities (p < 0.0001)- see S6 Fig.

### AI in refractive error prediction

Eight of 14 studies on RE prediction were included in the meta-analysis. The pooled sensitivity was 0.87 (95% CI: 0.73–0.94), specificity 0.96 (95% CI: 0.90–0.98), DOR 159.94 (95% CI: 40.17–636.85), and SROC 0.96 (95% CI: 0.85–0.95) as shown in .

**Fig 6:**
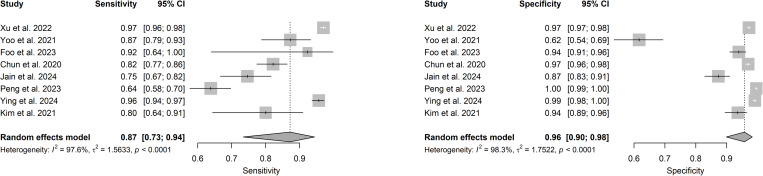
Forest plots for sensitivity (6A) and specificity (6B) for AI in refractive error prediction.

**Fig 7:**
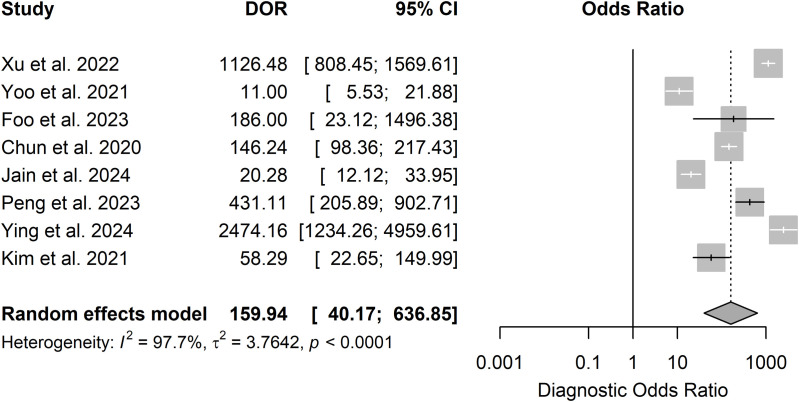
Forest plot for DOR for prediction studies.

**Fig 8:**
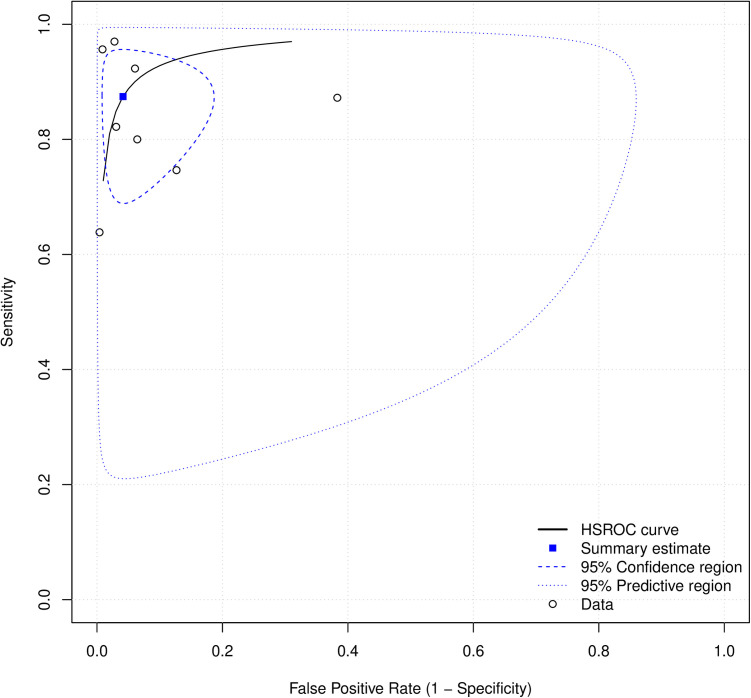
SROC curve for prediction studies.

### Threshold analysis for prediction studies

Threshold analysis showed no significant correlation (Spearman r = -0.119, p = 0.793), indicating heterogeneity among studies was unlikely due to differences in diagnostic thresholds.

### Publication bias for prediction studies

Egger’s funnel plot asymmetry test showed no publication bias (p = 0.495), as illustrated in S7 Fig).

### Subgroup analysis by AI technique for prediction studies

Five studies used DL as the backbone, with a pooled sensitivity of 0.89 (95% CI, 0.72–0.96), slightly outperforming the three studies using ML, which had a sensitivity of 0.84 (95% CI, 0.47–0.97); however, the difference was not statistically significant (p = 0.72). ML studies demonstrated higher pooled specificity (0.99, 95% CI, 0.93–1.00) and DOR (404.52, 95% CI, 53.21–3075.36) compared to DL studies (specificity 0.92, 95% CI, 0.79–0.97; DOR 91.07, 95% CI, 14.1–588.19), though these differences were also not statistically significant (p = 0.07 and p = 0.29, respectively) -see S8 Fig.

### Subgroup analysis by input modalities for prediction studies

Three studies used OCT images, two used eccentric photorefraction images, two used clinical data, and one study utilized a combination of fundus images and clinical data as input modalities. Eccentric photorefraction images demonstrated the highest pooled sensitivity at 0.92 (95% CI, 0.64–0.99), followed by the combination of fundus and clinical data at 0.92 (95% CI, 0.64–1.00), clinical data at 0.86 (95% CI, 0.34–0.99), and OCT images at 0.81 (95% CI, 0.71–0.88). However, studies using clinical data alone had the highest pooled specificity (0.99, 95% CI, 0.99–1.00) and DOR (1037.61, 95% CI, 187.24–5750.0). These values were superior to those observed in studies using eccentric photorefraction images (specificity: 0.97, DOR: 407.1), OCT images (specificity: 0.84, DOR: 22.08), and the combined modality (specificity: 0.94, DOR: 186.00). The differences in specificity and DOR among input modalities were statistically significant (p < 0.0001) -see S9 Fig.

### Review of AI in refractive error treatment

A total of 11 studies investigated the application of AI in the treatment of REs. Due to the limited number of eligible studies and the heterogeneity in reporting styles, a meta-analysis was not feasible. The studies primarily addressed areas such as orthokeratology (Ortho-K), laser refractive surgeries (including LASEK: Laser-Assisted Subepithelial Keratectomy, LASIK: Laser-Assisted In Situ Keratomileusis, and small lenticule incision extraction (SMILE)), myopia control outcomes, and implantable collamer lens (ICL) implantation. All studies utilized either clinical data (n = 8) or corneal topography maps (n = 3) as input modalities.

Tree-based ML algorithms, such as decision trees, random forest, XGBoost, CatBoost, and bagging trees, were the most applied modeling techniques. Other ML models included support vector machines (SVM), Gaussian process regression, Least Absolute Shrinkage and Selection Operator (LASSO) regression, and artificial neural networks (ANN). Among DL techniques, full convolutional networks, and innovative architectures like Segformer were used for image-based analysis.

Input features across studies frequently included demographic and biometric parameters such as age, axial length (AL), keratometry values (K1, K2), spherical equivalent refraction (SER), and white-to-white (WTW) distance. Target variables varied and encompassed treatment zone characteristics (e.g., alignment, curvature, diameter), axial length changes, effectiveness of myopia control, and lens design parameters (e.g., base curve, lens sag, optical zone diameter).

Several studies demonstrated strong predictive performance using AI models in the treatment of REs. For example, Li *et al*. employed bagging tree algorithms to predict corneal curvature parameters (K1 and K2) following ortho-k, achieving R values ranging from 0.812 to 0.837 and mean absolute errors (MAEs) between 0.669 and 0.701 [51]. Yang *et al*. utilized a deep neural network (DNN) to predict lens design parameters alignment curve (AC), treatment power (TP), and lens diameter (LD)—with excellent results (R² up to 0.97; mean squared error (MSE) between 0.01 and 0.08) [71]. Koo *et al.* [72] implemented multiple ML models, including CatBoost and Extra Trees, and achieved high classification accuracy for toric lens recommendations (accuracy = 0.927; F1-score = 0.929). They also reported strong regression performance for lens base curve prediction (R² = 0.948).In another study, Fang *et al.* reached a classification accuracy of 92.86% for predicting myopia control outcomes, with a sensitivity of 86.67% and specificity of 100% [50].

In the context of refractive surgery planning, several AI-based approaches have shown promising results. Yoo *et al*. applied the XGBoost algorithm to recommend appropriate surgical procedures, achieving an accuracy of 82.1% [76] while Yuan *et al.* utilized a backpropagation (BP) neural network to estimate lenticular thickness for SMILE surgery, reporting a MSE of 0.248 [86]. Advanced image-based segmentation techniques were also explored. Zhang *et al.* [69] and Tang *et a*l. [93] implemented DL architectures to delineate treatment zones on corneal topography maps with high precision—achieving a mean Intersection over Union (mIoU) of 97.19%, Dice Similarity Coefficient (DSC) of 0.94, and Intersection over Union (IoU) of 0.90 ± 0.06. For predicting postoperative refractive outcomes in ICL implantation, Jiang *et al.* [55] employed various ML models—including support vector regression, Random Forest, XGBoost, and LASSO regression. Using a comprehensive set of clinical parameters, they predicted postoperative SE and sphere values with strong accuracy, reporting MAEs of 0.339 diopters (D) for non-toric ICL (NT-ICL) and 0.325 D for toric ICL (TICL) lenses (see **Table 2**).

**Table 2:** Characteristics of studies for refractive error treatment.

Author, Year	Type of Images/Data Used	Input features/ (No.)	Treatment approach	AI Technique	Best model	Target variable	Performance metrics
Li *et al*. 2023 [51]	Corneal topography maps	Total number = 6 MTDP, K1after, K2after, K1after_axis, K2after_axis, SER	Ortho-K	ML	Bagging Tree	K1 and K2	K1 Model 1 R = 0.812, RMSE = 0.855, MAE = 0.671 K1 model 2 R = 0.812, RMSE = 0.858, MAE = 0.669 K2 Model 1 R = 0.831, RMSE = 0.898, MAE = 0.701 K2 Model 2 R = 0.837, RMSE = 0.888, MAE = 0.683
Jiang *et al*. 2023 [55]	Clinical data	Total number = 20 Age, sphere, cylinder, and cylinder axis, IOP, mesopic PD, scotopic PD, AL, K1, K2, K1 axis, K2 axis, ACA, ACD, CT, WTW, NT-ICL lens sphere, TICL lens sphere, cylinder and lens axis	ICL implantation	ML	Random forest	Postop- SE and sphere prediction of NT-ICL	NT-ICL-SE prediction MAE = 0.339D, SD = 0.445D, MedAE = 0.268D, Interquartile of AE = 0.372D NT-ICL-sphere prediction MAE = 0.386D, SD = 0.489D, MedAE = 0.336D, Interquartile of AE = 0.312D
					XGBoost	Postop- SE and sphere prediction of TICL	TICL-SE prediction MAE = 0.325D, SD = 0.452D, MedAE = 0.257D, Interquartile of AE = 0.316D TICL-sphere prediction MAE = 0.308D, SD = 0.443D, MedAE = 0.241D, Interquartile of AE = 0.344D
Zhang *et al*. 2024 [69]	Corneal topography maps	N/A	Ortho-K	DL	Segformer Architecture Network	Treatment Zone (TZ) Peripheral Steepened Zone (PSZ)	ACC = 99.03%, mIoU = 97.19%, mPA = 98.98%
Yang *et al*. 2024 [71]	Clinical data	Total number = 9 Flat K, Steep K, Corneal astigmatism, Flat, Steep e, E mean, BFS, Sagittal differential at 8 mm corneal zone, HVID	Ortho-K	DL	DNN	Alignment Curvature (AC) Target Power (TP) Lens Diameter (LD)	For AC, TP and LD MSE = 0.08 D, 0.07 D and 0.01 mm R² = 0.97 D, 0.95 D and 0.91 mm
Koo *et al*. 2024 [72]	Clinical data	Total number = 16 Age, AR Sph and AR Cyl, MR Sph and MR Cyl, flattest and steepest keratometry, mean keratometry, keratometry astigmatism, WTW, e value, flat e value, steep e value, axial length, CCT, ACD	Ortho-K	ML	CatBoost	Toric option RZD2 LZA	1. Toric lens: ACC = 0.927, Precision = 0.931, Recall = 0.927, F1 = 0.929 2. RZD2: MAE = 3.775 µm, RMSE = 11.703 µm, R² = 0.791 6. LZA: MAE = 0.121°, RMSE = 0.346°, R² = 0.798
					Extra Trees	OAD LensSag	1. OAD: ACC = 0.864, Precision = 0.868, Recall = 0.864, F1 = 0.865 2. LensSag:MAE = 4.372 µm, RMSE = 6.008 µm, R² = 0.921
					Least-angle regression	BC RZD1	1. BC: MAE = 0.054 µm, RMSE = 0.083µm, R² = 0.948 2. RZD1: MAE = 3.031 µm, RMSE = 8.574 µm, R² = 0.708
Yoo *et al*. 2020 [76]	Clinical data	Total number = 9 Corrected distance VA, Manifest refraction, Slit-lamp examination, and Dilated fundus examination, Corneal topography map, Central corneal thickness (CCT), Pupil size, NIBUT, Questionnaire survey	Refractive surgery	ML	Multiclass XGBoost	LASEK LASIK SMILE Contraindication	ACC = 82.1%
Fan *et al*. 2022 [77]	Clinical data	Total number = 8 Gender, Age, HVID, SER, e value, flat K (K1), steep K (K2), ACD, AL	Ortho-K	ML	Linear SVM	Steep K reading of AC1	AC1K1 R² = 0.91, MAE = 0.263, RMSE = 0.373, MSE = 0.139
					Gaussian process regression	Flat K reading of AC1 Flat K reading of AC2	AC1K2 R² = 0.84, MAE = 0.396, RMSE = 0.532, MSE = 0.283 AC2K1 R² = 0.73, MAE = 0.507, RMSE = 0.680, MSE = 0.462
Fang *et al*. 2023 [50]	Clinical data	Total number = 9 Age, Baseline AL, Pupil diameter, Lens wearing time, Time spent outdoors, Time spent on near work, WTW, Anterior corneal flat K, Posterior corneal astigmatism	Ortho-K	ML	LASSO regression	Myopia control effect of ortho-k	ACC = 92.86% SEN = 86.67% SPE = 100%
Xu *et al*. 2023 [78]	Clinical data	Total number = 9 Age of myopia onset, Number of myopic parents, Spherical power, cylindrical power, Flat K, Steep K, Corneal diameter, Eccentricity value, Baseline Axial length	Ortho-K	ML	Random forest	AC and TP Axial length after 1 year	R = 0.97 R² = 0.93 MAE = 0.185 MSE = 0.093
Yuan *et al*. 2023 [86]	Clinical data	Total number = 4 SPH, CYL, Km and lenticule diameter	SMILE surgery	ML	BP neural network	Lenticular thickness	MSE = 0.248 Gradient = 4.23
Tang *et al*. 2021 [93]	Corneal topography maps	Total number = 2 Axial subtractive maps, Tangential subtractive maps	Ortho-K	DL	Full convolutional network	Treatment zone boundary Treatment zone center	Treatment zone boundary IoU = 0.90 ± 0.06 DSC of 0.94 ± 0.04 Treat zone center Deviation = 6.32 ± 6.23 pixels

ML machine learning; DL Deep learning; DNN Deep neural network; SVM Support vector machine; Ortho-K Orthokeratology; K Keratometry; K1 Original flat K; K2 Original steep K; SER Spherical equivalent refraction; SE Spherical equivalent; AL Axial length; AR Autorefraction; MR Manifest refraction; ICL Implantable collamer lens; PD Pupillary distance; IOP Intraocular pressure; MTDP maximal tangential difference power; ACD Anterior chamber depth; ACA Anterior chamber depth; CT Corneal thickness; CCT Central corneal thickness; WTW white-to-white; NT-ICL non-toric ICL; TICL Toric-ICL; TZ Treatment Zone; PSZ Peripheral Steepened Zone; AC Alignment Curvature; TP Target Power; LD +Lens Diameter

### Review of progression of refractive error over a number of years

Among the included studies, six focused on predicting the progression of REs. Due to substantial heterogeneity in reporting styles and outcome measures, a meta-analysis was not feasible. These studies primarily investigated clinical outcomes related to RE progression trajectories, particularly the onset of myopia and high myopia. All six studies employed ML approaches, with commonly used algorithms including support vector machines (SVM), extreme gradient boosting (XGBoost), random forests, multivariate linear regression, and logistic regression. These models were trained on clinical datasets comprising input features such as age, SE, annual myopia progression rate, axial length-to-corneal radius (AL/CR) ratio, time intervals between baseline and follow-up visits, and SE at subsequent follow-ups [53,91,92]. XGBoost demonstrated superior performance in two studies [87,91]. For example, Li *et al*. reported an AUC of 0.96 for predicting annual myopia progression [87] while Zhao *et al.* achieved high predictive accuracy across multiple outcomes (R² = 0.613–0.992 for SE trajectory prediction and AUC = 0.845–0.953 for myopia onset) [91]. Another study by Li and colleagues achieved strong predictive performance using multivariate linear regression for SE trajectory (R² = 0.964, MAE = 0.119 D) and logistic regression for predicting high myopia onset (AUC = 0.99) [53]. In addition, Lin *et al.* employed random forest models to predict SE progression and high myopia development over a 10-year horizon, reporting AUC values ranging from 0.862 to 0.958 [92]. These results collectively underscore the potential of ML techniques in forecasting RE progression with high accuracy (summarized in **Table 3**).

**Table 3:** Characteristics of studies for predicting refractive error progression.

Author, Year	Data Used	Input features/ (No.)	AI Technique	Best model	Target variable	Performance metrics
Varosanec *et al*. 2024 [52]	Clinical data	Total number = 15 Date of first and follow-up visits, school-age group, gender, age, correction method, uncorrected VA, best-corrected VA, best-corrected VA binocularly, baseline cycloplegic SE, corrected SE, sphere, cylinder, and axis, parental myopia status, myopia classification	DL	Extended gate time-aware long short-term memory	Future SE	MAE = 0.10 ± 0.15 D
Li *et al*. 2024a [53]	Clinical data	Total number = 4 Age at baseline, SE at baseline, The time interval between baseline and follow-ups, Corresponding outputs: SE at subsequent follow-up sessions	ML	Multivariate linear regression	SE progression trajectory	R² = 0.964 MAE = 0.119D
				Logistic regression	High myopia onset	SEN = 98.96% SPE = 93.70% ACC = 94.31% AUC = 0.99
Barraza-Bernal *et al*. 2023 [84]	Clinical data	Total number = 3 SE, Age, AxL/Cr	ML	Support Vector Machines	Spherical power as a function of the age	R² = 0.57 RMSE = 1.33 D
Li *et al*. 2024b [87]	Clinical data	Total number = 1 SE	ML	XGBoost	Annual myopia progression	AUC = 0.96
Zhao *et al*. 2024 b [91]	Clinical data	Total number = 3 Age at baseline, SE at the first examination, Annual myopia progression rate	ML	XGBoost	1. SE over 15-year period 2. Onset of myopia 3. Onset of high myopia	R² = (0.613 -0.992) MAE = (0.078 - 1.673) MSE = (0.099 - 11.410) SEN = (0.853 -0.967) SPE = (0.53 - 0.986) ACC= (0.854 -0.971) AUC = (0.845 -0.953) SEN = (0.682-1.00) SPE = (0.804-0.994) ACC= (0.784-0.994) AUC = (0.765 - 0.997)
Lin *et al*. 2018 [92]	Clinical data	Total number = 3 Age at examination, SE, Annual progression rate	ML	Random forest	1. SE in 10 years 2. Presence of high myopia in 10 years	AUC 3 years = 0.903 to 0.958 5years = 0.886 to 0.889 8 years = 0.862 to 0.888 MAE 3 years = 0.253 to 0.395 5 years = 0.394 to 0.496 8 years = 0.503 to 0.799

SE: Spherical Equivalent; ML: Machine Learning; AxL/Cr: Axial Length/Corneal Radius ratio; VA Visual acuity; UCVA uncorrected visual acuity; BCVA Best corrected visual acuity; MAE: Mean Absolute Error; RMSE: Root Mean Square Error; SEN: Sensitivity; SPE: Specificity; ACC: Accuracy; AUC: Area Under the Curve

## Discussion

This study highlights the broad applications of AI technologies in the detection, diagnosis, prediction, progression, and treatment of REs. Overall, the findings suggest that AI holds significant potential to enhance the clinical management of REs in real-world settings, as demonstrated by consistently high sensitivity, specificity, and SROC values across various applications.

Our analysis demonstrated that AI, particularly, DL exhibited high accuracy in the detection and diagnosis of REs, with pooled SROC of 0.98, sensitivity of 95%, and specificity of 97%. This outstanding performance may be attributed to the established effectiveness of DL in ocular imaging tasks [94,95]. Deep learning models are especially advantageous due to their capacity to automatically extract and learn hierarchical and complex features from imaging modalities such as fundus photographs, thereby enhancing their utility in detection and diagnostic applications [96]. Another key finding from the meta-analysis of detection and diagnosis models was the significant impact of the input modality. Fundus images emerged as particularly effective, yielding an SROC greater than 0.98. This indicates that fundus photography offers reliable visual information essential for AI algorithms to accurately detect or diagnose REs, likely because it allows direct visualization of structural ocular changes associated with refractive conditions, particularly myopia [97]. Additionally, the robustness of DL performance may be partly attributed to the larger volume of studies employing this technique relative to other AI approaches, thereby offering more comprehensive evidence of its effectiveness.

In the prediction of REs, AI demonstrated high predictive performance, with a pooled SROC of 0.96 and pooled sensitivity and specificity of 87% and 96%, respectively. Our study found that DL models achieved a slightly higher sensitivity (0.89) compared to ML models (0.84). Conversely, ML models demonstrated higher specificity (0.99) compared to DL models (0.92), although these differences were not statistically significant. These results suggest that while DL models may be better at identifying positive cases, ML models may be more effective at reducing false positives, highlighting the importance of model selection based on specific clinical needs. It is important to note that the input data used by DL and ML models were not identical across the included studies. Notably, DL models in this category often utilized image-based modalities such as eccentric photorefraction images, which capture critical features relevant to refractive status, reinforcing the clinical utility of ocular appearance images in AI prediction. These findings indicate that AI, especially DL, could facilitate large-scale screening or early intervention, particularly in underserved or resource-limited settings where only ocular appearance images are available.

There has been growing interest in utilizing AI approaches to predict the progression of REs, particularly myopia and high myopia, over time. Nearly all the reviewed studies focusing on RE progression employed ML as their primary modeling technique. ML is well-suited for handling complex, multidimensional longitudinal patient data, especially in scenarios where clinical outcomes are not known in advance, enabling effective forecasting of disease trajectories [98,99]. We observed that all the included studies employed clinical features such as age, gender, parental myopia, and lifestyle factors like outdoor activity for predictive modeling—variables well-established as influencing myopia onset and progression [100,101]. Early identification of individuals exhibiting initial RE changes, particularly those at risk of developing high myopia, could facilitate timely therapeutic interventions aimed at slowing disease progression. This approach also supports personalized management strategies and better planning of future care. The AUC values reported across these progression studies ranged from 0.845 to 0.99, while R² values varied between 0.613 and 0.964, indicating that ML models have strong potential to accurately identify myopia progressors.

As demonstrated in the results section, multiple studies have proposed various DL and ML models for the treatment of REs, particularly focusing on predicting Ortho-K lens parameters, selecting optimal laser refractive surgery procedures, evaluating myopia control outcomes, and guiding ICL interventions. However, there was notable inconsistency in how results were reported across these studies. While many studies reported metrics such as R², MAE, and accuracy, this was not standardized. The reported R² values ranged from 0.708 to 0.97, accuracies varied between 82.1% and 99.03%, and MAE values spanned from 0.054D to 6.008D. These outcomes collectively underscore the precision and overall high performance of the AI models evaluated for treatment applications.

Of note, despite the comprehensiveness of our search strategy, we found no evidence of AI utilization in RE management across the African continent. This is particularly concerning given that Africa bears a substantial burden of UREs [102], and faces severe shortages of eye care professionals alongside significant challenges in delivering eye care services [103]. The marked scarcity of AI applications in RE management in this region highlights a significant healthcare disparity that urgently requires attention. In particular, this disparity can be attributed to multiple intersecting challenges, most notably the lack of high-quality, large-scale, and demographically diverse datasets from African populations. Most AI models in RE detection have been developed using datasets from high-income countries, often lacking representation of African ethnic and genetic diversity. As a result, such models may perform poorly when applied in African contexts, potentially leading to biased or inaccurate predictions [104–106]. Furthermore, clinical data collection systems in many African countries are either non-digitized or poorly maintained, with inadequate annotation, storage, and interoperability, limiting their usability for AI model training [104,107]. In addition to data limitations, there is a notable shortage of trained professionals in AI, data science, and medical image analysis across the continent [106,108]. Most healthcare workers and researchers have limited exposure to AI model development, validation, or implementation. Moreover, the few individuals who do receive AI training often face challenges such as inadequate infrastructure, limited access to GPUs, cloud computing services, and imaging technologies, further hindering innovation and local model development [107]. Beyond technical limitations, systemic barriers further complicate AI integration in African RE care. These include inadequate policy frameworks, limited funding for digital health innovation, ethical and privacy concerns, and low levels of community trust in AI technologies [109]. Language, cultural relevance, and the usability of AI tools also remain largely unexplored in current implementations, which are often designed for Western users and contexts. The adoption of advanced AI-based RE management systems could help bridge this gap by promoting equitable access to eye care services across Africa, thereby supporting the broader objectives of Sustainable Development Goal (SDG) 3 and the African Union’s Agenda 2063 vision for transformed healthcare systems. However, successful implementation of AI in African settings will likely require careful and context-specific planning, taking into account factors such as specialist availability, long-term patient outcomes, and the cost-effectiveness of integrating AI into existing healthcare infrastructures, especially compared to resource-rich countries. Specifically, a multi-faceted strategy is needed. Investments should focus on establishing robust data infrastructure, building AI capacity through education and cross-disciplinary training, and creating inclusive datasets that reflect the diversity of African populations. Partnerships between local institutions, international collaborators, and technology developers can facilitate sustainable and ethical AI integration. Such initiatives will be critical to ensuring that AI tools are not only technically sound but are also socially acceptable and clinically relevant in this region.

## Limitations

Although this study presents the first comprehensive evidence synthesis highlighting the utility of AI in RE management, some limitations warrant consideration. First, by restricting our review to English-language publications, relevant studies in other languages may have been excluded. Notably, over half of the included studies were conducted in Asia, which may reflect regional differences in RE prevalence. However, future research involving more diverse geographic and ethnic populations is necessary to better assess the real-world performance and generalizability of AI across different settings. While all studies performed internal validation, the lack of external validation raises concerns about the broader applicability of these models, underscoring the need for further validation efforts. Most studies employed retrospective designs, which are susceptible to selection bias [110], and there remains a paucity of high-quality prospective studies evaluating AI performance in real-time clinical environments. Current guidelines emphasize that regulatory approval for AI devices requires validation through multicenter randomized controlled trials using standardized methods to ensure reliability and clinical applicability [111]. Importantly, unlike RE detection, the long-term clinical benefits of AI, such as reducing the incidence and prevalence of REs, remain to be established, representing a critical outcome for future research [112].

## Conclusion

This systematic review and meta-analysis provide comprehensive evidence of the effectiveness and versatility of AI technologies, particularly DL and ML in the diagnosis, prediction, monitoring, and treatment of REs. Our findings indicate that AI models demonstrate high diagnostic accuracy, with pooled sensitivity and specificity exceeding 90%, particularly when using image-based inputs such as fundus photography. These results underscore the clinical potential of AI in improving early detection and intervention strategies for REs. In prediction and progression modeling, AI systems showed strong performance, with DL models favoring higher sensitivity and ML models offering better specificity. This suggests that model selection should be context-driven, depending on whether the goal is early case detection or minimizing false positives. Treatment-focused AI applications also showed promise, especially in supporting clinical decisions in orthokeratology, myopia control, and refractive surgeries, though variation in performance metrics and limited external validation call for cautious interpretation. A major strength of our review is its rigorous methodology, including adherence to PRISMA guidelines and the inclusion of a meta-analytic component. However, limitations such as reliance on English-language studies, retrospective designs, and a lack of studies from low-resource settings (particularly Africa) restrict generalizability. These gaps highlight significant disparities in AI adoption and the urgent need for inclusive datasets and equitable technological development. Future research should prioritize the development of interpretable, externally validated models trained on diverse, population-representative datasets. Strategic investments in data infrastructure, interdisciplinary collaboration, and digital capacity-building are critical, especially in underrepresented regions. Moreover, research should assess the real-world clinical impact, cost-effectiveness, and patient-centered outcomes of AI-assisted RE care. Taken together, AI holds transformative potential in RE management, and with responsible development and global inclusivity, it can serve as a scalable solution to address vision care challenges across diverse healthcare settings.

## Supporting information

S1 PRISMA Check list PRISMA ChecklistPRISMA (Preferred Reporting Items for Systematic Review and Meta-Analysis) checklist. (DOCX)

S2 Text The search strategy for each engine: a) PubMed, b) Web of Science, c) Embase, d) Scopus, e) Cochrane library and 5) Google scholar. (DOCX)

S3 Table Risk of bias of included studies using the Quality Assessment of Diagnostic Accuracy Studies-2 (QUADAS-2) tool. (XLSX)

S4 Fig Funnel plot asymmetry test for detection and diagnosis studies. (TIF)

S5 Fig Subgroup analysis plots by AI technique for detection and diagnosis studies. (TIF)

S6 Fig Subgroup analysis plots by input modalities for detection and diagnosis studies. (JPG)

S7 Fig Funnel plot asymmetry test for prediction studies. (TIF)

S8 Fig Subgroup analysis plots by AI technique for prediction studies. (JPG)

S9 Fig Subgroup analysis plots by input modalities for prediction studies. (JPG)

S10 File All studies identified in the literature search. (XLSX)

S11 File All data extracted from included studies. (XLSX)

S12 File Data used for meta-analysis. (XLSX)

## Abbreviations

AC: Alignment Curve
AL: Axial Length;
AL/CR: Axial Length-To-Corneal Radius Ratio
AI: Artificial Intelligence
ANN: Artificial Neural Network
AUC: Area Under The Curve
CCT: Central Corneal Thickness
CNN: Convolutional Neural Network
CR: Corneal Radius
DNN: Deep Neural Network
DL: Deep Learning
DOR: Diagnostic Odds Ratio
ICL: Implantable Collamer Lens
K1: Keratometry 1
K2: Keratometry 2
LASIK: Laser-Assisted In Situ Keratomileusis
LASEK: Laser-Assisted Subepithelial Keratectomy
LD: Lens Diameter
LASSO: Least Absolute Shrinkage and Selection Operator
MAE: Mean Absolute Error
MSE: Mean Squared Error
ML: Machine Learning
OCT: Optical Coherence Tomography
Ortho-K: Orthokeratology
PRISMA: Preferred Reporting Items for Systematic Review and Meta-Analysis
R²: Coefficient of Determination
SE: Spherical Equivalent
SER: Spherical Equivalent Refraction
SMILE: Small Incision Lenticule Extraction
SROC: Summary Receiver Operating Characteristic
SVM: Support Vector Machine
TP: Treatment Power
URE: Uncorrected Refractive Error
WTW: White-to-White Distance
XGBoost: Extreme Gradient Boosting. 2023

## References

[1] Pascolini D, Mariotti SP. Global estimates of visual impairment: 2010. Br J Ophthalmol. 2012;96(5):614–8. doi: 10.1136/bjophthalmol-2011-300539 22133988

[2] Harb EN, Wildsoet CF. Origins of refractive errors: Environmental and genetic factors. Annual Review of Vision Science. 2019;5(5):47–72. 10.1146/annurev-vision-091718-015027 PMC11827892 31525141

[3] Grosvenor T, Grosvenor TP. Primary care optometry. Elsevier Health Sciences. 2007.

[4] WHO. Blindness and vision impairment. https://www.who.int/news-room/fact-sheets/detail/blindness-and-visual-impairment. 2021. 2022.

[5] Naidoo K, Gichuhi S, Basáñez M-G, Flaxman SR, Jonas JB, Keeffe J, et al. Prevalence and causes of vision loss in sub-Saharan Africa: 1990-2010. Br J Ophthalmol. 2014;98(5):612–8. doi: 10.1136/bjophthalmol-2013-304081 24568870

[6] Holden B, Davis S, Jong M, Resnikoff S. The evolution of uncorrected refractive error as a major public health issue. Journal and Proceedings of the Royal Society of New South Wales. 2014.

[7] Flaxman SR, Bourne RR, Resnikoff S, Ackland P, Braithwaite T, Cicinelli MV. Global causes of blindness and distance vision impairment 1990–2020: a systematic review and meta-analysis. The Lancet Global Health. 2017;5(12):e1221–34. 10.1016/S2214-109X(17)30393-5 29032195

[8] WHO. Report of the 2030 targets on effective coverage of eye care. 2022: World Health Organization.

[9] Kemchoknatee P, Sunlakaviset P, Khieokhoen N, Srisombut T, Tangon D. A comparison of autorefraction and subjective refraction in an academic optometry clinic. Cureus, 2023. 15(4). 10.7759/cureus.37448 PMC10174683 37182059

[10] Atchison DA. Objective refraction. Optometry: science, techniques and clinical management. 2016;13:187–208.

[11] García-Guerra CE, Martínez-Roda JA, Ondategui-Parra JC, Turull-Mallofré A, Aldaba M, Vilaseca M. System for Objective Assessment of the Accommodation Response During Subjective Refraction. Transl Vis Sci Technol. 2023;12(5):22. doi: 10.1167/tvst.12.5.22 37219508 PMC10210517

[12] Rosenfield M. Subjective refraction. Optometry: Science, Techniques and Clinical Management. 2nd ed. Edinburgh: Butterworth Heinemann/Elsevier. 2009. 209–28.

[13] Chaitra M, Harshitha K, Kumar HM, Archana S. Subjective verification of refraction-a quality section indicator: a cross-sectional study. Journal of clinical and diagnostic research. 2023;17(6):NC06–8.

[14] Aggarwal A, Gairola S, Upadhyay U, Vasishta AP, Rao D, Goyal A, et al. Towards automating retinoscopy for refractive error diagnosis. Proceedings of the ACM on Interactive, Mobile, Wearable and Ubiquitous Technologies. 2022;6(3):Article 97.

[15] Rodriguez-Lopez V, Dorronsoro C. Beyond traditional subjective refraction. Curr Opin Ophthalmol. 2022;33(3):228–34. doi: 10.1097/ICU.0000000000000834 35102097

[16] Zhang C, Zhao J, Zhu Z, Li Y, Li K, Wang Y, , et al. Applications of artificial intelligence in myopia: current and future directions. Frontiers in Medicine. 2022;9. 10.3389/fmed.2022.840498 PMC8962670 35360739

[17] Holden BA, Fricke TR, Wilson DA, Jong M, Naidoo KS, Sankaridurg P, et al. Global prevalence of myopia and high myopia and temporal trends from 2000 through 2050. Ophthalmology. 2016;123(5):1036–42. 26875007 10.1016/j.ophtha.2016.01.006

[18] Poole DL, Mackworth AK. Artificial Intelligence: Foundations of Computational Agents. Cambridge University Press. 2010.

[19] Ertel W. Introduction to artificial intelligence. Springer. 2018.

[20] Buiten MC. Towards intelligent regulation of artificial intelligence. European Journal of Risk Regulation. 2019;10(1):41–59.

[21] Du X-L, Li W-B, Hu B-J. Application of artificial intelligence in ophthalmology. Int J Ophthalmol. 2018;11(9):1555–61. doi: 10.18240/ijo.2018.09.21 30225234 PMC6133903

[22] Matheny ME, Whicher D, Israni ST. Artificial intelligence in health care: a report from the National Academy of Medicine. JAMA. 2020;323(6):509–10. 31845963 10.1001/jama.2019.21579

[23] Grassmann F, Mengelkamp J, Brandl C, Harsch S, Zimmermann ME, Linkohr B, et al. A Deep Learning Algorithm for Prediction of Age-Related Eye Disease Study Severity Scale for Age-Related Macular Degeneration from Color Fundus Photography. Ophthalmology. 2018;125(9):1410–20. doi: 10.1016/j.ophtha.2018.02.037 29653860

[24] Peng Y, Dharssi S, Chen Q, Keenan TD, Agrón E, Wong WT, et al. DeepSeeNet: A Deep Learning Model for Automated Classification of Patient-based Age-related Macular Degeneration Severity from Color Fundus Photographs. Ophthalmology. 2019;126(4):565–75. doi: 10.1016/j.ophtha.2018.11.015 30471319 PMC6435402

[25] Tham Y-C, Goh JHL, Anees A, Lei X, Rim TH, Chee M-L, et al. Detecting visually significant cataract using retinal photograph-based deep learning. Nat Aging. 2022;2(3):264–71. doi: 10.1038/s43587-022-00171-6 37118370 PMC10154193

[26] Tham Y-C, Anees A, Zhang L, Goh JHL, Rim TH, Nusinovici S, et al. Referral for disease-related visual impairment using retinal photograph-based deep learning: a proof-of-concept, model development study. Lancet Digit Health. 2021;3(1):e29–40. doi: 10.1016/S2589-7500(20)30271-5 33735066

[27] Gulshan V, Peng L, Coram M, Stumpe MC, Wu D, Narayanaswamy A, et al. Development and Validation of a Deep Learning Algorithm for Detection of Diabetic Retinopathy in Retinal Fundus Photographs. JAMA. 2016;316(22):2402–10. doi: 10.1001/jama.2016.17216 27898976

[28] Ting DSW, Cheung CY-L, Lim G, Tan GSW, Quang ND, Gan A, et al. Development and Validation of a Deep Learning System for Diabetic Retinopathy and Related Eye Diseases Using Retinal Images From Multiethnic Populations With Diabetes. JAMA. 2017;318(22):2211–23. doi: 10.1001/jama.2017.18152 29234807 PMC5820739

[29] Liu H, et al. Development and validation of a deep learning system to detect glaucomatous optic neuropathy using fundus photographs. JAMA Ophthalmology. 2019;137(12):1353–60. 31513266 10.1001/jamaophthalmol.2019.3501 PMC6743057

[30] Kapoor R, Walters SP, Al-Aswad LA. The current state of artificial intelligence in ophthalmology. Surv Ophthalmol. 2019;64(2):233–40. doi: 10.1016/j.survophthal.2018.09.002 30248307

[31] Ying B, Chandra RS, Wang J, Cui H, Oatts JT. Machine learning models for predicting cycloplegic refractive error and myopia status based on non-cycloplegic data in Chinese students. Translational Vision Science & Technology. 2024;13(8):16. 10.1167/tvst.13.8.16 PMC11318358 39120886

[32] Choi KJ, Choi JE, Roh HC, Eun JS, Kim JM, Shin YK, et al. Deep learning models for screening of high myopia using optical coherence tomography. Sci Rep. 2021;11(1):21663. doi: 10.1038/s41598-021-00622-x 34737335 PMC8568935

[33] Li M, Liu S, Wang Z, Li X, Yan Z, Zhu R et al. MyopiaDETR: End-to-end pathological myopia detection based on transformer using 2D fundus images. Front NeuroSci . 2023. 10.3389/fnins.2023.1130609 PMC9941630 36824210

[34] Xu D, Ding S, Zheng T, Zhu X, Gu Z, Ye B et al. Deep learning for predicting refractive error from multiple photorefraction images. BioMedical Engineering. 2022. 10.1186/s12938-022-01025-3 PMC9360706 35941613

[35] Zhao J, Yu Y, Li Y, Li F, Zhang Z, Jian W, et al., Development and validation of predictive models for myopia onset and progression using extensive 15-year refractive data in children and adolescents. Journal of Translational. 2024: Springer. 10.1186/s12967-024-05075-0 PMC10946190 38494492

[36] Gong J, Li K, Hu J, Chen C, Chen H, Cao Q, Wu G, et al, Application of machine learning method in clinical fitting of Ortho-K lens for myopia correction. 2022: www.researchsquare.com

[37] Lu L, hou E, Yu W, Chen B, Ren P, Lu Q, et al Development of deep learning-based detecting systems for pathologic myopia using retinal fundus images. Communications. 2021: www.nature.com 10.1038/s42003-021-02758-y PMC8548495 34702997

[38] Kim YC, Chang DJ, Park SJ, Choi IY, Gong YS, Kim H-A, et al. Machine learning prediction of pathologic myopia using tomographic elevation of the posterior sclera. Sci Rep. 2021;11(1):6950. doi: 10.1038/s41598-021-85699-0 33772040 PMC7997908

[39] Kundu G, Virani I, Shetty R, Khamar P, Nuijts RMMA. Role of artificial intelligence in determining factors impacting patients’ refractive surgery decisions. Indian J Ophthalmol. 2023;71(3):810–7. doi: 10.4103/IJO.IJO_2718_22 36872684 PMC10229918

[40] Shen J, et al. Artificial intelligence versus clinicians in disease diagnosis: systematic review. JMIR Medical Informatics. 2019;7(3):e10010. 10.2196/10010 PMC6716335 31420959

[41] Alnahedh TA, Taha M. Role of Machine Learning and Artificial Intelligence in the Diagnosis and Treatment of Refractive Errors for Enhanced Eye Care: A Systematic Review. Cureus. 2024;16(4). 10.7759/cureus.57706 PMC11071623 38711688

[42] Moher D, Liberati A, Tetzlaff J, Altman DG, PRISMA Group. Preferred reporting items for systematic reviews and meta-analyses: the PRISMA statement. Ann Intern Med. 2009;151(4):264–9, W64. doi: 10.7326/0003-4819-151-4-200908180-00135 19622511

[43] Higgins J, et al. Cochrane handbook for systematic reviews of interventions version 6.4 (updated August 2023). Cochrane. 2023.

[44] Page MJ, McKenzie JE, Bossuyt PM, Boutron I, Hoffmann TC, Mulrow CD. The PRISMA 2020 statement: an updated guideline for reporting systematic reviews. BMJ. 2021;372. 10.1136/bmj.n71 PMC8005924 33782057

[45] Babineau J. Product review: Covidence (systematic review software). Journal of the Canadian Health Libraries Association/Journal de l’Association des bibliothèques de la santé du Canada. 2014;35(2):68–71.

[46] Whiting PF, Rutjes AWS, Westwood ME, Mallett S, Deeks JJ, Reitsma JB, et al. QUADAS-2: a revised tool for the quality assessment of diagnostic accuracy studies. Ann Intern Med. 2011;155(8):529–36. doi: 10.7326/0003-4819-155-8-201110180-00009 22007046

[47] Higgins JPT, Thompson SG, Deeks JJ, Altman DG. Measuring inconsistency in meta-analyses. BMJ. 2003;327(7414):557–60. doi: 10.1136/bmj.327.7414.557 12958120 PMC192859

[48] Peng W, Wang F, Sun S, Sun Y, Chen J, Wang M. Does multidimensional daily information predict the onset of myopia? A 1-year prospective cohort study. BioMedical Engineering. 2023. 10.1186/s12938-023-01109-8 PMC10182351 37179307

[49] Barraza-Bernal MJ, Ohlendorf A, Diez PS, Feng X, Yang LH, Lu MX, et al. Prediction of refractive error and its progression: a machine learning-based algorithm. BMJ Open Ophthalmology. 2023;8(1). 10.1136/bmjophth-2023-001298 PMC10551949 37793703

[50] Fang J, et al. Machine learning for predicting the treatment effect of orthokeratology in children. Frontiers in Pediatrics. 2023;10:1057863. 36683821 10.3389/fped.2022.1057863 PMC9853046

[51] Li Y, Zhao H, Fan Y, Hu J, Li S, Wang K, et al. A machine learning-based algorithm for estimating the original corneal curvature based on corneal topography after orthokeratology. Cont Lens Anterior Eye. 2023;46(4):101862. doi: 10.1016/j.clae.2023.101862 37208285

[52] Varošanec AM, Marković L, Sonicki Z. A Novel Time-Aware Deep Learning Model Predicting Myopia in Children and Adolescents. Ophthalmol Sci. 2024;4(6):100563. doi: 10.1016/j.xops.2024.100563 39165695 PMC11334700

[53] Li J, Zeng S, Li Z, Xu J, Sun Z, Zhao J, et al. Accurate prediction of myopic progression and high myopia by machine learning. Precis Clin Med. 2024;7(1):pbae005. doi: 10.1093/pcmedi/pbae005 38558949 PMC10981449

[54] Lu L, Ren P, Tang X, Yang M, Yuan M, Yu W, et al. AI-Model for Identifying Pathologic Myopia Based on Deep Learning Algorithms of Myopic Maculopathy Classification and “Plus” Lesion Detection in Fundus Images. Front Cell Dev Biol. 2021;9:719262. doi: 10.3389/fcell.2021.719262 34722502 PMC8554089

[55] Jiang Y, Shen Y, Chen X, Niu L, Li B, Cheng M, et al. Artificial intelligence-based refractive error prediction and EVO-implantable collamer lens power calculation for myopia correction. Eye and Vision. 2023;10(1):22. 37121995 10.1186/s40662-023-00338-1 PMC10150472

[56] Pathan S, Siddalingaswamy P, Dsouza N. Automated detection of pathological and non-pathological myopia using retinal features and dynamic ensemble of classifiers. Telecommunications and Radio Engineering. 2020;79(20).

[57] Zhang Z, Xu Y, Liu J, Wong DWK, Kwoh CK, Saw S-M, et al. Automatic diagnosis of pathological myopia from heterogeneous biomedical data. PLoS One. 2013;8(6):e65736. doi: 10.1371/journal.pone.0065736 23799040 PMC3683061

[58] Zhang Z, Xu Y, Liu J, Wong DWK, Kwoh CK, Saw S-M, et al. Automatic diagnosis of pathological myopia from heterogeneous biomedical data. PLoS One. 2013;8(6):e65736. doi: 10.1371/journal.pone.0065736 23799040 PMC3683061

[59] Peng H, Li J, Cheng W, Zhao L, Guan Y, Li Z, et al. Automatic diagnosis of pediatric high myopia via Attention-based Patch Residual Shrinkage network. Expert Systems with Applications. 2024;255:124704. doi: 10.1016/j.eswa.2024.124704

[60] Yang Y, Li R, Lin D, Zhang X, Li W, Wang J, et al. Automatic identification of myopia based on ocular appearance images using deep learning. Ann Transl Med. 2020;8(11):705. doi: 10.21037/atm.2019.12.39 32617325 PMC7327333

[61] Linde G, Chalakkal R, Zhou L, Huang JL, O’Keeffe B, Shah D, et al. Automatic Refractive Error Estimation Using Deep Learning-Based Analysis of Red Reflex Images. Diagnostics (Basel). 2023;13(17):2810. doi: 10.3390/diagnostics13172810 37685347 PMC10486607

[62] Xu D, Ding S, Zheng T, Zhu X, Gu Z, Ye B, et al. Deep learning for predicting refractive error from multiple photorefraction images. Biomed Eng Online. 2022;21(1):55. doi: 10.1186/s12938-022-01025-3 35941613 PMC9360706

[63] Varadarajan AV, Poplin R, Blumer K, Angermueller C, Ledsam J, Chopra R, et al. Deep Learning for Predicting Refractive Error From Retinal Fundus Images. Invest Ophthalmol Vis Sci. 2018;59(7):2861–8. doi: 10.1167/iovs.18-23887 30025129

[64] Yoo TK, Ryu IH, Kim JK, Lee IS. Deep learning for predicting uncorrected refractive error using posterior segment optical coherence tomography images. Eye. 2022;36(10):1959–65. 34611313 10.1038/s41433-021-01795-5 PMC9500028

[65] Park S-J, Ko T, Park C-K, Kim Y-C, Choi I-Y. Deep Learning Model Based on 3D Optical Coherence Tomography Images for the Automated Detection of Pathologic Myopia. Diagnostics (Basel). 2022;12(3):742. doi: 10.3390/diagnostics12030742 35328292 PMC8947335

[66] Choi KJ, Choi JE, Roh HC, Eun JS, Kim JM, Shin YK, et al. Deep learning models for screening of high myopia using optical coherence tomography. Sci Rep. 2021;11(1):21663. doi: 10.1038/s41598-021-00622-x 34737335 PMC8568935

[67] Foo LL, Lim GYS, Lanca C, Wong CW, Hoang QV, Zhang XJ, et al. Deep learning system to predict the 5-year risk of high myopia using fundus imaging in children. NPJ Digit Med. 2023;6(1):10. doi: 10.1038/s41746-023-00752-8 36702878 PMC9879938

[68] Chun J, Kim Y, Shin KY, Han SH, Oh SY, Chung T-Y, et al. Deep Learning-Based Prediction of Refractive Error Using Photorefraction Images Captured by a Smartphone: Model Development and Validation Study. JMIR Med Inform. 2020;8(5):e16225. doi: 10.2196/16225 32369035 PMC7238094

[69] Zhang M, Guo Y, Zhou C, Zhang J, Zhang M, Huang J, et al. Deep neural network with self-attention based automated determination system for treatment zone and peripheral steepened zone in Orthokeratology for adolescent myopia. Cont Lens Anterior Eye. 2024;47(1):102081. doi: 10.1016/j.clae.2023.102081 37957085

[70] Jain R, Yoo TK, Ryu IH, Song J, Kolte N, Nariani A. Deep Transfer Learning for Ethnically Distinct Populations: Prediction of Refractive Error Using Optical Coherence Tomography. Ophthalmol Ther. 2024;13(1):305–19. doi: 10.1007/s40123-023-00842-6 37955835 PMC10776546

[71] Yang H-WW, Liang C-KL, Chou S-C, Wang H-H, Chiang HK. Development and evaluation of a deep neural network model for orthokeratology lens fitting. Ophthalmic Physiol Opt. 2024;44(6):1224–36. doi: 10.1111/opo.13360 38980216

[72] Koo S, Kim WK, Park YK, Jun K, Kim D, Ryu IH, et al. Development of a Machine-Learning-Based Tool for Overnight Orthokeratology Lens Fitting. Transl Vis Sci Technol. 2024;13(2):17. doi: 10.1167/tvst.13.2.17 38386347 PMC10896231

[73] Lu L, Zhou E, Yu W, Chen B, Ren P, Lu Q, et al. Development of deep learning-based detecting systems for pathologic myopia using retinal fundus images. Commun Biol. 2021;4(1):1225. doi: 10.1038/s42003-021-02758-y 34702997 PMC8548495

[74] Peng W, Wang F, Sun S, Sun Y, Chen J, Wang M. Does multidimensional daily information predict the onset of myopia? A 1-year prospective cohort study. Biomed Eng Online. 2023;22(1):45. doi: 10.1186/s12938-023-01109-8 37179307 PMC10182351

[75] Ren P-F, Tang X-Y, Yu C-Y, Zhu L-L, Yang W-H, Shen Y. Evaluation of a novel deep learning based screening system for pathologic myopia. Int J Ophthalmol. 2023;16(9):1417–23. doi: 10.18240/ijo.2023.09.07 37724265 PMC10475629

[76] Yoo TK, et al. Explainable machine learning approach as a tool to understand factors used to select the refractive surgery technique on the expert level. Translational Vision Science & Technology. 2020;9(2):8. 10.1167/tvst.9.2.8 PMC7346876 32704414

[77] Fan Y, Yu Z, Tang T, Liu X, Xu Q, Peng Z, et al. Machine learning algorithm improves accuracy of ortho-K lens fitting in vision shaping treatment. Cont Lens Anterior Eye. 2022;45(3):101474. doi: 10.1016/j.clae.2021.101474 34301476

[78] Xu S, Yang X, Zhang S, Zheng X, Zheng F, Liu Y, et al. Machine learning models for orthokeratology lens fitting and axial length prediction. Ophthalmic Physiol Opt. 2023;43(6):1462–8. doi: 10.1111/opo.13212 37574762

[79] Kim YC, Chang DJ, Park SJ, Choi IY, Gong YS, Kim H-A, et al. Machine learning prediction of pathologic myopia using tomographic elevation of the posterior sclera. Sci Rep. 2021;11(1):6950. doi: 10.1038/s41598-021-85699-0 33772040 PMC7997908

[80] Huang J, Ma W, Li R, Zhao N, Zhou T. Myopia prediction for children and adolescents via time-aware deep learning. Sci Rep. 2023;13(1):5430. doi: 10.1038/s41598-023-32367-0 37012269 PMC10070443

[81] Zhao J, Cao G, He J, Dai C. Multi-class classification of pathological myopia based on fundus photography. Journal of Innovative Optical Health Sciences. 2024;17(06):2450016.

[82] Hemelings R, Elen B, Blaschko MB, Jacob J, Stalmans I, De Boever P. Pathological myopia classification with simultaneous lesion segmentation using deep learning. Comput Methods Programs Biomed. 2021;199:105920. doi: 10.1016/j.cmpb.2020.105920 33412285

[83] Yang X, Chen G, Qian Y, Wang Y, Zhai Y, Fan D, et al. Prediction of Myopia in Adolescents through Machine Learning Methods. Int J Environ Res Public Health. 2020;17(2):463. doi: 10.3390/ijerph17020463 31936770 PMC7013571

[84] Barraza-Bernal MJ, et al. Prediction of refractive error and its progression: a machine learning-based algorithm. BMJ Open Ophthalmology. 2023;8(1). 10.1136/bmjophth-2023-001298 PMC10551949 37793703

[85] Yang D, Li M, Li W, Wang Y, Niu L, Shen Y, et al. Prediction of refractive error based on ultrawide field images with deep learning models in myopia patients. Frontiers in Medicine. 2022;9. 10.3389/fmed.2022.834281 PMC9007166 35433763

[86] Yuan D-Q, Tang F-N, Yang C-H, Zhang H, Wang Y, Zhang W-W, et al. Prediction of SMILE surgical cutting formula based on back propagation neural network. Int J Ophthalmol. 2023;16(9):1424–30. doi: 10.18240/ijo.2023.09.08 37724263 PMC10475637

[87] Li W, Tu Y, Zhou L, Ma R, Li Y, Hu D, et al. Study of myopia progression and risk factors in Hubei children aged 7-10 years using machine learning: a longitudinal cohort. BMC Ophthalmol. 2024;24(1):93. doi: 10.1186/s12886-024-03331-x 38429630 PMC10905806

[88] Zhu S, Zhan H, Yan Z, Wu M, Zheng B, Xu S, et al. Prediction of spherical equivalent refraction and axial length in children based on machine learning. Indian J Ophthalmol. 2023;71(5):2115–31. doi: 10.4103/IJO.IJO_2989_22 37203092 PMC10391375

[89] Tan T-E, Anees A, Chen C, Li S, Xu X, Li Z, et al. Retinal photograph-based deep learning algorithms for myopia and a blockchain platform to facilitate artificial intelligence medical research: a retrospective multicohort study. Lancet Digit Health. 2021;3(5):e317–29. doi: 10.1016/S2589-7500(21)00055-8 33890579

[90] Ali S, Raut S. Smartphone app to detect pathological myopia using spatial attention and squeeze-excitation network as a classifier and segmentation encoder. International Journal of Imaging Systems and Technology. 2024;34(5):e23157.

[91] Zhao J, Yu Y, Li Y, Li F, Zhang Z, Jian W, et al. Development and validation of predictive models for myopia onset and progression using extensive 15-year refractive data in children and adolescents. J Transl Med. 2024;22(1):289. doi: 10.1186/s12967-024-05075-0 38494492 PMC10946190

[92] Lin H, Long E, Ding X, Diao H, Chen Z, Liu R, et al. Prediction of myopia development among Chinese school-aged children using refraction data from electronic medical records: A retrospective, multicentre machine learning study. PLoS Med. 2018;15(11):e1002674. doi: 10.1371/journal.pmed.1002674 30399150 PMC6219762

[93] Tang Y, Chen Z, Wang W, Wen L, Zhou L, Wang M, et al. A Deep Learning-Based Framework for Accurate Evaluation of Corneal Treatment Zone After Orthokeratology. Transl Vis Sci Technol. 2021;10(14):21. doi: 10.1167/tvst.10.14.21 34932118 PMC8709934

[94] Yoo TK, Choi JY. Outcomes of Adversarial Attacks on Deep Learning Models for Ophthalmology Imaging Domains. JAMA Ophthalmol. 2020;138(11):1213–5. doi: 10.1001/jamaophthalmol.2020.3442 33001161 PMC7530819

[95] De Fauw J, Ledsam JR, Romera-Paredes B, Nikolov S, Tomasev N, Blackwell S, et al. Clinically applicable deep learning for diagnosis and referral in retinal disease. Nat Med. 2018;24(9):1342–50. doi: 10.1038/s41591-018-0107-6 30104768

[96] Poplin R, Varadarajan AV, Blumer K, Liu Y, McConnell MV, Corrado GS, et al. Prediction of cardiovascular risk factors from retinal fundus photographs via deep learning. Nat Biomed Eng. 2018;2(3):158–64. doi: 10.1038/s41551-018-0195-0 31015713

[97] Du R, Ohno-Matsui K. Novel Uses and Challenges of Artificial Intelligence in Diagnosing and Managing Eyes with High Myopia and Pathologic Myopia. Diagnostics (Basel). 2022;12(5):1210. doi: 10.3390/diagnostics12051210 35626365 PMC9141019

[98] Ngufor C, Van Houten H, Caffo BS, Shah ND, McCoy RG. Mixed effect machine learning: A framework for predicting longitudinal change in hemoglobin A1c. J Biomed Inform. 2019;89:56–67. doi: 10.1016/j.jbi.2018.09.001 30189255 PMC6495570

[99] Du W, Cheung H, Goldberg I, Thambisetty M, Becker K, Johnson CA. A Longitudinal Support Vector Regression for Prediction of ALS Score. IEEE Int Conf Bioinform Biomed Workshops. 2015;2015:1586–90. doi: 10.1109/BIBM.2015.7359912 27042700 PMC4814169

[100] Pärssinen O, Kauppinen M, Viljanen A. The progression of myopia from its onset at age 8–12 to adulthood and the influence of heredity and external factors on myopic progression. A 23-year follow-up study. Acta Ophthalmologica. 2014;92(8):730–9. 24674576 10.1111/aos.12387

[101] Theophanous C, Modjtahedi BS, Batech M, Marlin DS, Luong TQ, Fong DS. Myopia prevalence and risk factors in children. Clin Ophthalmol. 2018;12:1581–7. doi: 10.2147/OPTH.S164641 30214142 PMC6120514

[102] Sherwin JC, Lewallen S, Courtright P. Blindness and visual impairment due to uncorrected refractive error in sub-Saharan Africa: review of recent population-based studies. Br J Ophthalmol. 2012;96(7):927–30. doi: 10.1136/bjophthalmol-2011-300426 22317912

[103] Courtright P, Seneadza A, Mathenge W, Eliah E, Lewallen S. Primary eye care in sub-Saharan African: do we have the evidence needed to scale up training and service delivery?. Ann Trop Med Parasitol. 2010;104(5):361–7. doi: 10.1179/136485910X12743554760225 20819303

[104] Kolawole E, D O DO. Impact and challenges of artificial intelligence integration in the African health sector: A review. Trends in Medical Research. 2024;19:220–35.

[105] Ibeneme S, Karamagi H, Muneene D, Goswami K, Chisaka N, Okeibunor J. Strengthening Health Systems Using Innovative Digital Health Technologies in Africa. Front Digit Health. 2022;4:854339. doi: 10.3389/fdgth.2022.854339 35434700 PMC9008130

[106] Arakpogun EO, Elsahn Z, Olan F, Elsahn F. Artificial intelligence in Africa: Challenges and opportunities. The fourth industrial revolution: Implementation of artificial intelligence for growing business success. 2021. 375–88.

[107] Ade-Ibijola A, Okonkwo C. Artificial intelligence in Africa: Emerging challenges. Responsible AI in Africa: Challenges and opportunities. Cham: Springer International Publishing. 2023. 101–17.

[108] Coker MO, et al. Data science training needs in sub-Saharan Africa: implications for biomedical research and therapeutics capacity. Open Research Africa. 2023;6:21.

[109] Owoyemi A, Owoyemi J, Osiyemi A, Boyd A. Artificial intelligence for healthcare in Africa. Frontiers in Digital Health. 2020;2:6. 34713019 10.3389/fdgth.2020.00006 PMC8521850

[110] Powell JT, Sweeting MJ. Retrospective Studies. Eur J Vasc Endovasc Surg. 2015;50(5):675. doi: 10.1016/j.ejvs.2015.07.005 26251354

[111] Lam TYT, Cheung MFK, Munro YL, Lim KM, Shung D, Sung JJY. Randomized Controlled Trials of Artificial Intelligence in Clinical Practice: Systematic Review. J Med Internet Res. 2022;24(8):e37188. doi: 10.2196/37188 35904087 PMC9459941

[112] Yang WH, Shao Y, Xu YW. Guidelines on clinical research evaluation of artificial intelligence in ophthalmology. Int J Ophthalmol. 2023;16(9):1361–72. 37724285 10.18240/ijo.2023.09.02 PMC10475621

