# Supplementary material for: Artificial intelligence applications in refractive error management: A systematic review and meta-analysis
Source: PLOS Digit Health. 2025 Sep 25;4(9):e0000904. doi: 10.1371/journal.pdig.0000904 (PMC12463214; doi:10.1371/journal.pdig.0000904)
Supplement: S2 Text — (DOCX) [file pdig.0000904.s002.docx]

**Full search terms of our review**

We show the search strategy for a) PubMed b) Web of science c) Embase d) Scopus e) Cochrane library f) Google Scholar

1. PubMed = 746

| Search | Query | Results |
| --- | --- | --- |
| #1 | ((((((((((((((((refractive error[Title/Abstract]) OR (refractive disorder[Title/Abstract])) OR (refractive anomaly[Title/Abstract])) OR (ametropia[Title/Abstract])) OR (myopia[Title/Abstract])) OR (nearsightedness[Title/Abstract])) OR (near-sightedness[Title/Abstract])) OR (shortsightedness[Title/Abstract])) OR (short-sightedness[Title/Abstract])) OR (hyperopia[Title/Abstract])) OR (hypermetropia[Title/Abstract])) OR (farsightedness[Title/Abstract])) OR (far-sightedness[Title/Abstract])) OR (longsightedness[Title/Abstract])) OR (long-sightedness[Title/Abstract])) OR (astigmatism[Title/Abstract])) OR (spherical equivalent[Title/Abstract])) | 42,594 |
| #2 | (((refractive error[MeSH Terms]) OR (myopia[MeSH Terms])) OR (hyperopia[MeSH Terms])) OR (astigmatism[MeSH Terms]) | 40,127 |
| #3 | (#1) OR (#2) | 56,83 |
| #4 | (((((((((((((((((((((((((((((artificial intelligence[Title/Abstract]) OR (AI[Title/Abstract])) OR (machine learning[Title/Abstract])) OR (deep learning[Title/Abstract])) OR (ensemble learning[Title/Abstract])) OR (machine intelligence[Title/Abstract])) OR (neural net*[Title/Abstract])) OR (convolutional neural net*[Title/Abstract])) OR (recurrent neural*[Title/Abstract])) OR (deep neural net*[Title/Abstract])) OR (artificial neural net*[Title/Abstract])) OR (transfer learning[Title/Abstract])) OR (computer vision[Title/Abstract])) OR (large language model*[Title/Abstract])) OR (hierarchical learning[Title/Abstract])) OR (algorithm[Title/Abstract])) OR (image analysis[Title/Abstract])) OR (pattern recognition[Title/Abstract])) OR (ResNet[Title/Abstract])) OR (GoogLeNet[Title/Abstract])) OR (EfficientNet[Title/Abstract])) OR (DenseNet[Title/Abstract])) OR (Random forest[Title/Abstract])) OR (K-NearestNeighbor[Title/Abstract])) OR (VGGNet[Title/Abstract])) OR (Support vector machine[Title/Abstract])) OR (gradient boosting[Title/Abstract])) OR (XGBoost[Title/Abstract])) OR (decision tree[Title/Abstract])) OR (Naive bayesian[Title/Abstract]) | 694,287 |
| #5 | ((((artificial intelligence[MeSH Terms]) OR (machine learning[MeSH Terms])) OR (deep learning[MeSH Terms])) OR (neural network[MeSH Terms])) OR (computer vision[MeSH Terms]) | 224,075 |
| #6 | (#4) OR (#5) | 772,811 |
| #7 | (((((diagnos*[Title/Abstract]) OR (detect*[Title/Abstract])) OR (predict*[Title/Abstract])) OR (progression[Title/Abstract])) OR (treatment[Title/Abstract])) OR (management[Title/Abstract]) | 9,666,031 |
| #8 | ((#3) AND (#6)) AND (#7) | 746 |

1. Web of Science = 2,802

| #1 | TS=(refractive error OR refractive disorder OR refractive anomaly OR ametropia OR myopia OR nearsightedness OR near-sightedness OR shortsightedness OR short-sightedness OR hyperopia OR hypermetropia OR farsightedness OR far-sightedness OR longsightedness OR long-sightedness OR astigmatism OR spherical equivalent) | 74,600 |
| --- | --- | --- |
| #2 | TS=(artificial intelligence OR AI OR machine learning OR deep learning OR ensemble learning OR machine intelligence OR neural net* OR convolutional neural net* OR recurrent neural net* OR deep neural net* OR artificial neural net* OR transfer learning OR computer vision OR large language model* OR hierarchical learning OR algorithm OR image analysis OR pattern recognition OR ResNet OR GoogLeNet OR EfficientNet OR DenseNet OR random forest OR K-Nearest Neighbor OR VGGNet OR support vector machine OR SVM OR gradient boosting OR XGBoost OR decision tree OR naive bayesian) | 5,265,383 |
| #3 | TS=(diagnos* OR detect* OR predict* OR progression OR treatment OR management) | 21,162,783 |
| #4 | #1 AND #2 AND #3 and Article (Document Types) | 2,802 |

1. Embase = 532

| #1 | 'refractive error':ab,ti OR 'refractive disorder':ab,ti OR 'refractive anomaly':ab,ti OR ametropia:ab,ti OR myopia:ab,ti OR nearsightedness:ab,ti OR 'near sightedness':ab,ti OR shortsightedness:ab,ti OR 'short sight*':ab,ti OR hyperopia:ab,ti OR hypermetropia:ab,ti OR farsightedness:ab,ti OR 'far sightedness':ab,ti OR longsightedness:ab,ti OR 'long sightedness':ab,ti OR astigmatism:ab,ti OR 'spherical equivalent':ab,ti | 54,217 |
| --- | --- | --- |
| #2 | 'artificial intelligence':ab,ti OR ai:ab,ti OR 'machine learning':ab,ti OR 'deep learning':ab,ti OR 'ensemble learning':ab,ti OR 'machine intelligence':ab,ti OR 'neural net*':ab,ti OR 'convolutional neural net*':ab,ti OR 'recurrent neural net*':ab,ti OR 'deep neural net*':ab,ti OR 'artificial neural net*':ab,ti OR 'transfer learning':ab,ti OR 'computer vision':ab,ti OR 'large language model*':ab,ti OR 'hierarchical learning':ab,ti OR 'algorithm':ab,ti OR 'image analysis':ab,ti OR 'pattern recognition':ab,ti OR 'resnet':ab,ti OR 'googlenet':ab,ti OR 'efficientnet':ab,ti OR 'densenet':ab,ti OR 'random forest':ab,ti OR 'k nearest neighbor':ab,ti OR 'vggnet':ab,ti OR 'support vector machine':ab,ti OR 'svm':ab,ti OR 'gradient boosting':ab,ti OR 'xgboost':ab,ti OR 'decision tree':ab,ti OR 'naive bayesian':ab,ti | 839,093 |
| #3 | 'diagnos*':ab,ti OR 'detect*':ab,ti OR 'predict*':ab,ti OR 'progression':ab,ti OR 'treatment':ab,ti OR 'management':ab,ti | 17,018,739 |
| #4 | #1 AND #2 AND #3 AND [article]/lim | 532 |

1. Scopus = 1,623

| #1 | TITLE-ABS-KEY ( "refractive error" OR "refractive disorder" OR "refractive anomaly" OR ametropia OR myopia OR nearsightedness* OR "near sightedness*" OR shortsightedness* OR "short sightedness*" OR hyperopia OR hypermetropia OR farsightedness* OR "far sightedness*" OR longsightedness* OR "long sightedness*" OR astigmatism OR "spherical equivalent" ) | 83,650 |
| --- | --- | --- |
| #2 | TITLE-ABS-KEY ( "artificial intelligence" OR ai OR "machine learning" OR "deep learning" OR "ensemble learning" OR "machine intelligence" OR "neural net*" OR "convolutional neural net*" OR "recurrent neural net*" OR "deep neural net*" OR "artificial neural net*" OR "transfer learning" OR "computer vision" OR "large language model*" OR "hierarchical learning" OR algorithm OR "image analysis" OR "pattern recognition" OR resnet OR googlenet OR efficientnet OR densenet OR "random forest" OR "K-Nearest Neighbor" OR vggnet OR "support vector machine" OR svm OR "gradient boosting" OR xgboost OR "decision tree" OR "Naive Bayesian" ) | 6,754,220 |
| #3 | TITLE-ABS-KEY ( diagnos* OR detect* OR predict* OR progression OR treatment OR management ) | 29,420,231 |
| #4 | #1 AND #2 AND #3 AND ( LIMIT-TO ( DOCTYPE , "ar" ) ) AND ( LIMIT-TO ( LANGUAGE , "English" ) ) | 1,623 |

1. Cochrane library = 105

| #1 | "refractive error" OR "refractive disorder" OR "refractive anomaly" OR ametropia OR myopia OR nearsightedness OR "near sightedness" OR shortsightedness OR "short sightedness" OR hyperopia OR hypermetropia OR farsightedness OR "far sightedness" OR longsightedness OR "long sightedness" OR astigmatism OR "spherical equivalent" | 6661 |
| --- | --- | --- |
|  | "artificial intelligence" OR ai OR "machine learning" OR "deep learning" OR "ensemble learning" OR "machine intelligence" OR "neural network" OR "convolutional neural network" OR "recurrent neural network" OR "deep neural network" OR "artificial neural network" OR "transfer learning" OR "computer vision" OR "large language model" OR "hierarchical learning" OR algorithm OR "image analysis" OR "pattern recognition" OR resnet OR googlenet OR efficientnet OR densenet OR "random forest" OR "K-Nearest Neighbor" OR vggnet OR "support vector machine" OR "gradient boosting" OR xgboost OR "Decision tree" OR "Naive Bayesian" | 37051 |
| #3 | diagnos* OR detect* OR predict* OR progression OR treatment OR management | 1,321,883 |
| #4 | #1 AND #2 AND #3 in Trials | 105 |

1. Google scholar = 480 (first 24 pages)

(refractive error OR refractive disorder OR refractive anomaly OR ametropia OR myopia OR nearsightedness OR near-sightedness OR shortsightedness OR short-sightedness OR hyperopia OR hypermetropia OR farsightedness OR farsightedness OR long sightedness OR long-sightedness OR astigmatism OR spherical equivalent) AND (artificial intelligence OR AI OR machine learning OR deep learning OR ensemble learning OR machine intelligence OR neural net* OR convolutional neural net* OR recurrent neural net* OR deep neural net* OR artificial neural net* OR transfer learning OR computer vision OR large language model* OR hierarchical learning OR algorithm OR image analysis OR pattern recognition OR ResNet OR GoogLeNet OR EfficientNet OR DenseNet OR random forest OR K-Nearest Neighbor OR VGGNet OR support vector machine OR SVM OR gradient boosting OR XGBoost OR decision tree OR naive bayesian) AND (diagnosis* OR detect* OR predict* OR progression OR treatment OR management)
