# Supplementary figures and images for: Artificial intelligence applications in refractive error management: A systematic review and meta-analysis
Source: PLOS Digit Health. 2025 Sep 25;4(9):e0000904. doi: 10.1371/journal.pdig.0000904 (PMC12463214; doi:10.1371/journal.pdig.0000904)

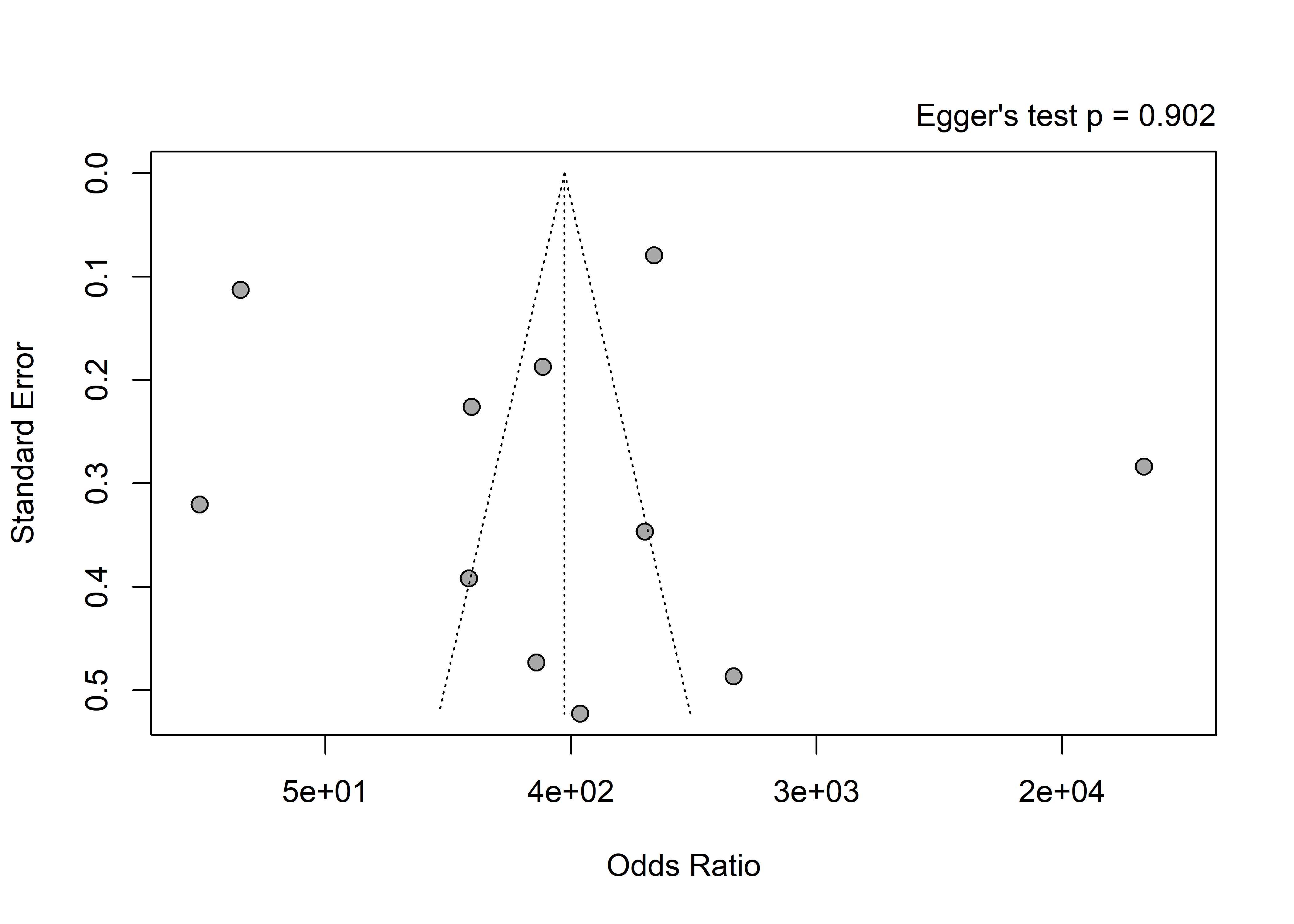

Supplement: S4 Fig — (TIF) [file pdig.0000904.s004.tif]

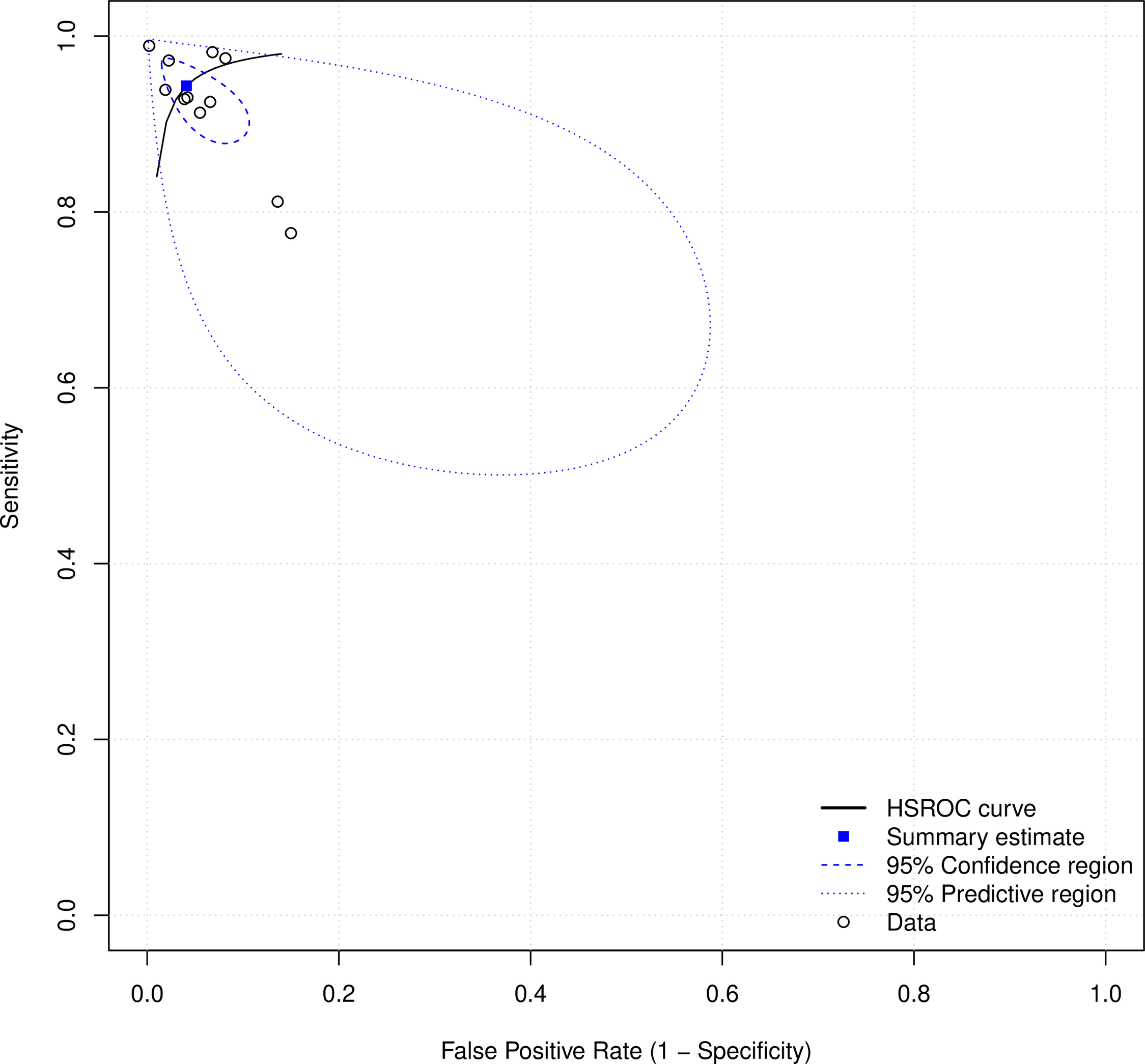

Supplement: S5 Fig — (TIF) [file pdig.0000904.s005.tif]

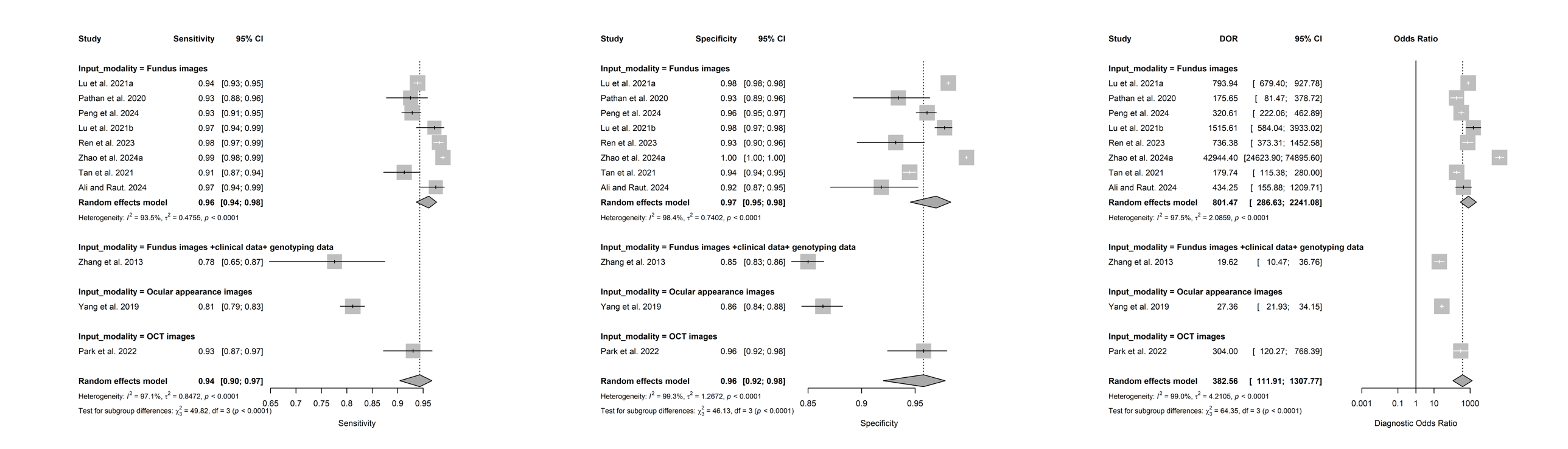

Supplement: S6 Fig — (JPG) [file pdig.0000904.s006.jpg]

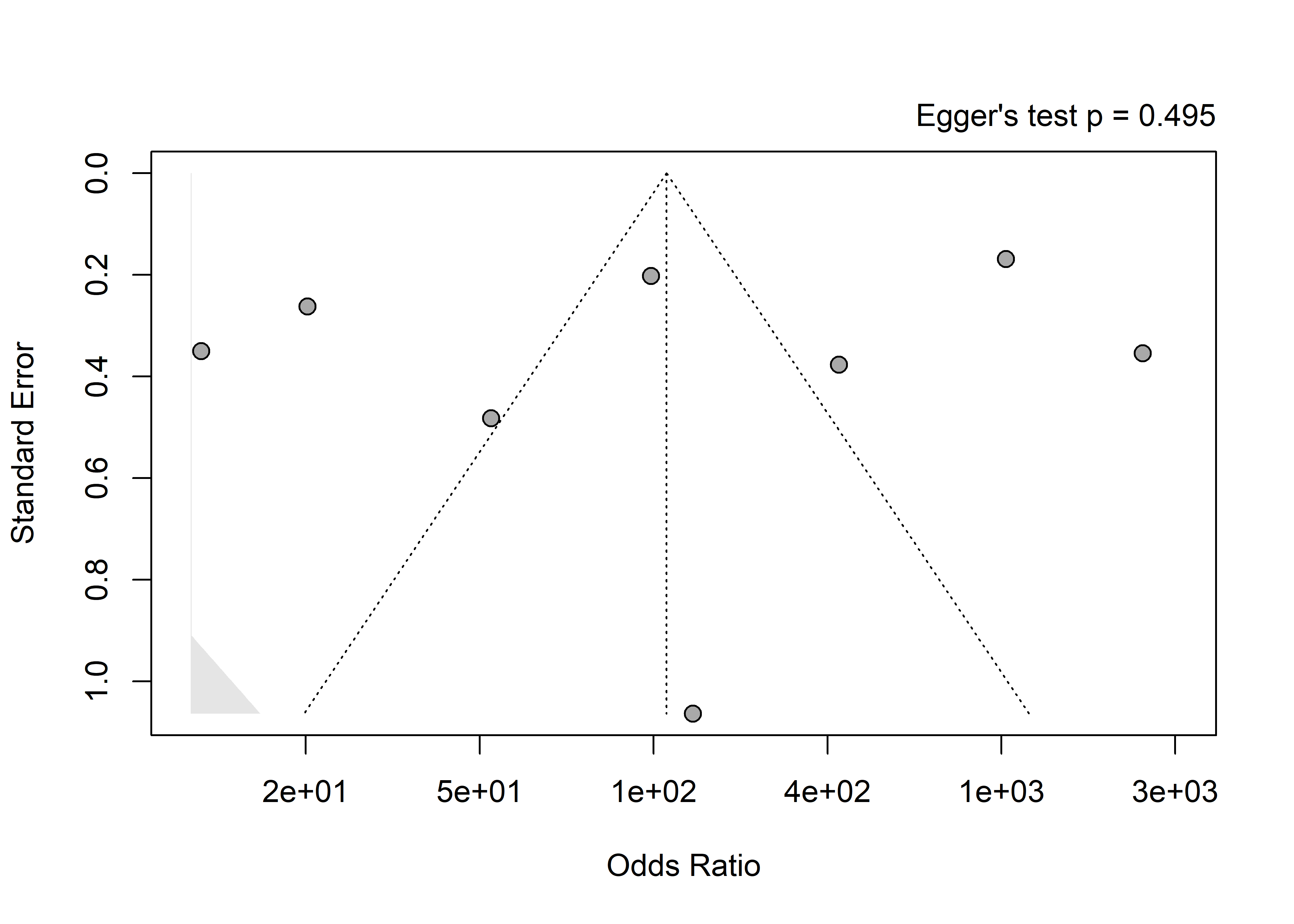

Supplement: S7 Fig — (TIF) [file pdig.0000904.s007.tif]

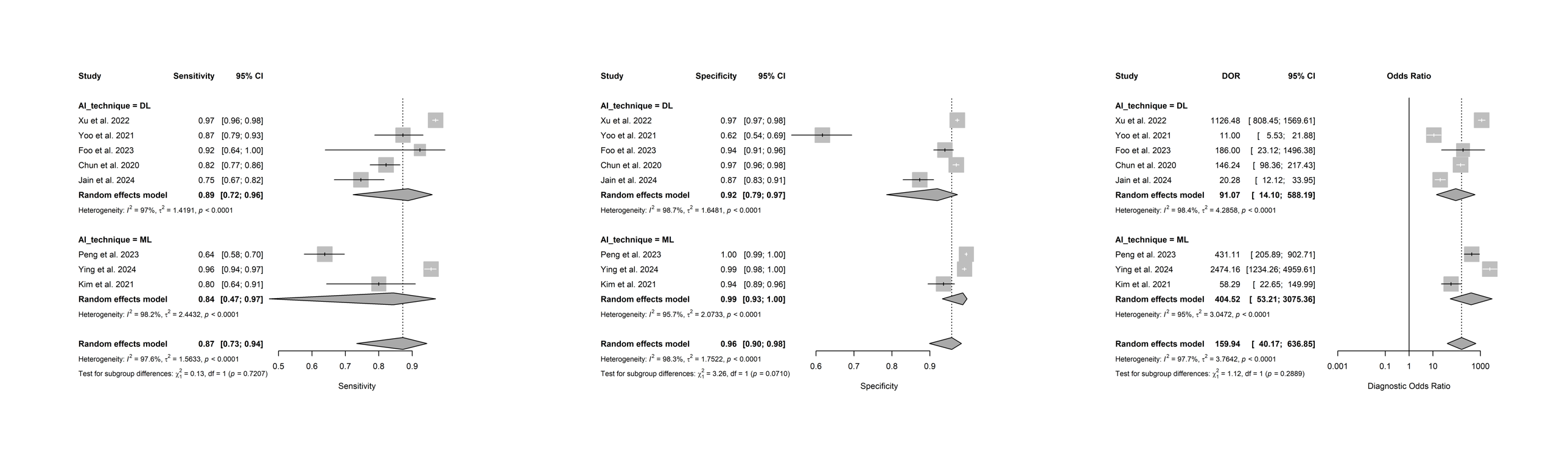

Supplement: S8 Fig — (JPG) [file pdig.0000904.s008.jpg]

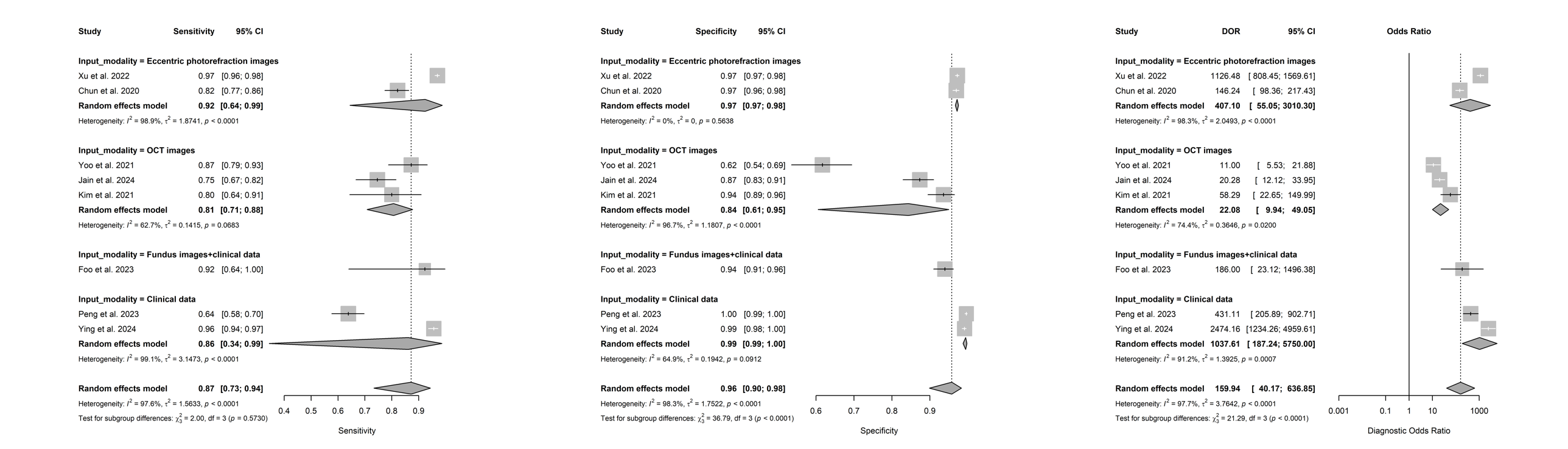

Supplement: S9 Fig — (JPG) [file pdig.0000904.s009.jpg]
