# Supplementary material for: Artificial intelligence applications in refractive error management: A systematic review and meta-analysis
Source: PLOS Digit Health. 2025 Sep 25;4(9):e0000904. doi: 10.1371/journal.pdig.0000904 (PMC12463214; doi:10.1371/journal.pdig.0000904)
Supplement: Attachment — Submitted filename: Response to editorial assistant_KOA_05212025.pdf [file pdig.0000904.s013.pdf]

Dr. Kate Anne Manalo Dimayuga

Editorial Assistant

PLOS Digital Health

Dear Dr. Kate,

PDIG-D-25-00300: Artificial intelligence applications in refractive error management: A systematic review and meta-analysis

Thank you for the opportunity to submit our above reference manuscript. We have addressed all queries point by point below and the revised changes made on the editorial submission system. All the comments presented by the editorial assistant is in black normal font and we have responded directly in a bold black font.

1. Your manuscript is a Systematic or Scoping Review. Please change the article type to 'Research Article', and ensure all submission questions are completed, noting the change in publication fees associated with Research Articles.

Thank you for pointing this out. We have changed from Systematic review to Research article in the editorial management system.

2. Where possible, we encourage authors to provide relevant data underlying their research with their submission in order to facilitate editorial and peer review, even if the data may be restricted from publication or made private by a data repository prior to article acceptance. Many repositories permit private access for review purposes, and have policies for public release at publication. For data concerning human research subjects, please anonymize the data as appropriate and follow pertinent legal and ethical protocols prior to sharing with editors and reviewers. If you are unable to share any pertinent data at the time of editorial and peer review, please kindly let the journal office know.

Thank you for highlighting this point. We have added a data availability statement to our manuscript as seen below and in the revised manuscript.

Data availability

This is a systematic review and data generated are all secondary. All data are included in the manuscript and supporting files.

3. We note that this manuscript is a systematic review or meta-analysis; our author guidelines require that you upload a PRISMA checklist as supporting information. Information about the PRISMA guidance and blank checklists can be found here: http://www.prisma-statement.org/. Please note that if your study analyzes clinical trials, we cannot proceed with consideration of your article until we have this information.

We have added a filled PRISMA statement delineating the components of our manuscript and its fit with the individual checklist.
