# Supplementary material for: Artificial intelligence applications in refractive error management: A systematic review and meta-analysis
Source: PLOS Digit Health. 2025 Sep 25;4(9):e0000904. doi: 10.1371/journal.pdig.0000904 (PMC12463214; doi:10.1371/journal.pdig.0000904)
Supplement: Attachment — Submitted filename: Response to Reviewers Comments_KOA_08082025.pdf [file pdig.0000904.s014.pdf]

PDIG-D-25-00300

Artificial intelligence applications in refractive error management: A systematic review and meta-analysis

PLOS Digital Health

Dear Dr. Leo,

Thank you for the opportunity to resubmit the above-referenced manuscript. We have addressed all the Reviewers' comments, point-by-point, in our response below. The issues raised by the Reviewers are presented in normal font. We reply directly to these comments in bold font. All changes to the Revised Manuscript have also been highlighted in track changes.

**We have uploaded the following files in our resubmission:**

*** A rebuttal letter that responds to each point raised by the editor and reviewer(s).**

*** A marked-up copy of your manuscript that highlights changes made to the original version.**

*** An unmarked version of your revised paper without tracked changes.**

**Sincerely,**

**Dr Kwadwo Owusu Akuffo (OD, PhD), on behalf of the authors.**

Journal Requirements:

1. Please upload separate figure files in .tif or .eps format. Also, remove the figures from your manuscript file but keep the legends.

For more information about figure files please see our guidelines:

https://journals.plos.org/digitalhealth/s/figures

https://journals.plos.org/digitalhealth/s/figures#loc-file-requirements

**We have uploaded separate figure files in the recommended format.**

2. Please provide an Author Summary. This should appear in your manuscript between the Abstract (if applicable) and the Introduction, and should be 150–200 words long. The aim should be to make your findings accessible to a wide audience that includes both scientists and non-scientists. Sample summaries can be found on our website under Submission Guidelines:

https://journals.plos.org/digitalhealth/s/submission-guidelines#loc-parts-of-a-submission

**We have provided an Author Summary as shown below and in the revised marked manuscript, Page 3, Lines 58-72.**

***"Author Summary***

***Refractive errors—such as nearsightedness, farsightedness, and astigmatism—are common vision problems that affect people of all ages. They can usually be corrected with glasses, contact lenses, or surgery, but early and accurate detection is key to effective treatment. In recent years, artificial intelligence (AI) has become a valuable tool in improving how these conditions are diagnosed, monitored, and managed. We investigate how AI is being used to detect, predict, track progression, and help treat these conditions. We analyzed 45 studies, of which 19 were included in a detailed meta-analysis. Over half used deep learning, a type of AI that mimics the human brain, while the others used machine learning. We found that AI tools were very accurate at diagnosing and predicting refractive errors, with high sensitivity (correctly identifying those with the condition) and specificity (correctly identifying those without it). AI was also useful in tracking changes in vision over time and in helping guide treatment choices, although performance varied more in those areas. Overall, AI shows strong potential to improve how we detect and manage refractive errors. Future work should focus on developing AI systems that work well across different populations and clinical settings."***

3. As required by our policy on Data Availability, please ensure your manuscript or supplementary information includes the following:

A numbered table of all studies identified in the literature search, including those that were excluded from the analyses.

For every excluded study, the table should list the reason(s) for exclusion.

**We have provided a table for all studies identified in the literature search**

If any of the included studies are unpublished, include a link (URL) to the primary source or detailed information about how the content can be accessed.

**No unpublished study was included in the review**

A table of all data extracted from the primary research sources for the systematic review and/or meta-analysis. The table must include the following information for each study:

Name of data extractors and date of data extraction

Confirmation that the study was eligible to be included in the review.

**We have added a table for all data extracted from included studies**

All data extracted from each study for the reported systematic review and/or meta-analysis that would be needed to replicate your analyses.

**We have provided the data used for analyses**

If data or supporting information were obtained from another source (e.g. correspondence with the author of the original research article), please provide the source of data and dates on which the data/information were obtained by your research group.

**We did not obtain data from correspondence that was included in this review.**

If applicable for your analysis, a table showing the completed risk of bias and quality/certainty assessments for each study or outcome. Please ensure this is provided for each domain or parameter assessed. For example, if you used the Cochrane risk-of-bias tool for randomized trials, provide answers to each of the signaling questions for each study. If you used GRADE to assess certainty of evidence, provide judgements about each of the quality of evidence factor. This should be provided for each outcome.

**We have provided the table showing the risk of bias**

An explanation of how missing data were handled.

Page 7, Lines 198-199: We have provided an explanation of how missing data were handled

**"Only records with sufficient information for primary outcomes were included. Missing data were not imputed."**

This information can be included in the main text, supplementary information, or relevant data repository. Please note that providing these underlying data is a requirement for publication in this journal, and if these data are not provided your manuscript might be rejected.

4. We note that your Data Availability Statement is currently as follows:

**[All relevant data and materials supporting the conclusion of this article is/are available within the manuscript and its supporting information files]**

Please confirm at this time whether or not your submission contains all raw data required to replicate the results of your study. Authors must share the "minimal data set" for their submission. PLOS defines the minimal data set to consist of the data required to replicate all study findings reported in the article, as well as related metadata and methods (https://journals.plos.org/plosone/s/data-availability#loc-minimal-data-set-definition).

For example, authors should submit the following data:

- The values behind the means, standard deviations and other measures reported;
- The values used to build graphs;
- The points extracted from images for analysis.

Authors do not need to submit their entire data set if only a portion of the data was used in the reported study.

If your submission does not contain these data, please either upload them as Supporting Information files or deposit them to a stable, public repository and provide us with the relevant

URLs, DOIs, or accession numbers. For a list of recommended repositories, please see https://journals.plos.org/plosone/s/recommended-repositories.

If there are ethical or legal restrictions on sharing a de-identified data set, please explain them in detail (e.g., data contain potentially sensitive information, data are owned by a third-party organization, etc.) and who has imposed them (e.g., an ethics committee). Please also provide contact information for a data access committee, ethics committee, or other institutional body to which data requests may be sent. If data are owned by a third party, please indicate how others may request data access.

**We have uploaded all dataset accompanying this manuscript as supporting information.**

If the reviewer comments include a recommendation to cite specific previously published works, please review and evaluate these publications to determine whether they are relevant and should be cited. There is no requirement to cite these works unless the editor has indicated otherwise.

**This is not applicable.**

Additional Editor Comments (if provided):

[Note: HTML markup is below. Please do not edit.]

Reviewers' Comments:

Reviewer #1: Line 43 of the abstract states that the work is "drawn from a pool of 8.086 studies". However, this does not match the PRISMA diagram (Figure 1), which shows 6,288 articles were retrieved. Which is the correct number?

**Page 2, Line 42: The correct number is 6,288. We have updated the number in the abstract.**

**"A total of 45 studies were included in the systematic review, drawn from a pool of 6,288 studies"**

Similarly, also starting on line 43, "Nineteen of these studies were permissible for meta-analysis." But the PRISMA diagram (Figure 1) shows that 45 studies were selected for inclusion. Which is the correct number? Or, is this mixing literature review with meta-analysis? If so, please make this clearer in the manuscript. Based on line 158, I suspect this is true.

**Page 5, Fig 1: We have indicated in the PRISMA diagram that the total number of included studies is 45 and the number of studies included in the quantitative analysis is 19.**

The PRISMA diagram (Figure 1) is referred to in line 155 (results) but it would make more sense to be referred to in the method.

**Page 4, Lines 151: We have referenced Figure 1 in the Methods (under study selection).**

**"Study selection and database flow were documented using the PRISMA flow diagram (Fig 1)(45)."**

Referring to the above, what criteria enabled studies to be included in the review but prevented them from being included in the meta-analysis? This should be included in the method.

**Page 7, Lines 197-198: We have added the specific criteria for meta-analysis eligibility.**

**"Studies were eligible for meta-analysis if they provided sufficient data to construct 2×2 diagnostic tables, or reported sensitivity and specificity values."**

There are some minor grammatical errors; please carefully proofread the manuscript. Similarly, there are some minor typing errors; sometimes there is a space before the citation [1], and sometimes there is not[1].

**The manuscript has been carefully proofread and revised to correct all such issues, including citation formatting and grammar.**

Line 69 states, "Traditional methods for diagnosing and managing refractive errors rely on subjective assessments". However, autorefraction tests are now fairly common. This doesn't rely heavily on practitioner expertise or patient input. However. Autorefraction testing is not mentioned in this manuscript.

**Page 3, Line 99-104: We have revised the sentence to acknowledge autorefraction as a widely used objective method in current clinical practice.**

**"Traditional methods for diagnosing and managing REs rely on several eye tests such as visual acuity testing [9], objective refraction [10] and subjective refraction [11]. Objective techniques such as autorefraction and retinoscopy offer quicker, more standardized alternatives. However, autorefraction, especially without cycloplegia, is prone to overestimating myopia or underestimating hyperopia due to accommodation, while retinoscopy - though accurate, requires technically skilled personnel, which may not be feasible during high-volume screening."**

It may be worth visualising some of the study characteristics reported from line 166 and AI applications from line 176.

**Page 14: We have added one visualization on the types of AI techniques and input modalities used in the included studies.**

Some abbreviations, such as Machine Learning (ML), are defined multiple times.

**We have revised the manuscript to define each abbreviation only at first mention and ensured consistency throughout.**

In Table 1, I am not convinced that "Main Outcome" is the most appropriate title for that column. Perhaps something like target prediction, model aim, or something else.

**Page 9, Table 1: The column title has been changed to *"Model Aim"* to better reflect its content.**

In Tables 2 and 3, is "Best model" the correct column header? Some rows have more than one model. What dataset were these works evaluated on? Were they all evaluated on the same dataset and using the same methodology? If not, then the direct comparison in the performance metrics column may not make sense.

**Thank you for pointing this out. However, for all studies in Tables 2 and 3, we extracted performance metrics data from the best model among all models used on the same dataset with the same methodology. To clarify this, we retained the *"Best Model"* column and replaced the *"outcome variable"* column with *"Target Variable,"* to specify the outcome each best model addressed, along with their performance metrics.**

The conclusion should be strengthened to better summarise the key findings and their implications. You may also wish to highlight any strengths or weaknesses of your manuscript. Similarly, there is an opportunity to further expand on the suggested future works.

**We have provided a detailed summary of the conclusion with thoughtful consideration of the reviewers comments as shown below and in the revised manuscript. Conclusion, Pages 33-34, lines 904 to 930.**

**"Conclusion**

**This systematic review and meta-analysis provide comprehensive evidence of the effectiveness and versatility of AI technologies, particularly DL and ML in the diagnosis, prediction, monitoring, and treatment of REs. Our findings indicate that AI models demonstrate high diagnostic accuracy, with pooled sensitivity and specificity exceeding 90%, particularly when using image-based inputs such as fundus photography. These results underscore the clinical potential of AI in improving early detection and intervention**

**strategies for REs. In prediction and progression modeling, AI systems showed strong performance, with DL models favoring higher sensitivity and ML models offering better specificity. This suggests that model selection should be context-driven, depending on whether the goal is early case detection or minimizing false positives. Treatment-focused AI applications also showed promise, especially in supporting clinical decisions in orthokeratology, myopia control, and refractive surgeries, though variation in performance metrics and limited external validation call for cautious interpretation. A major strength of our review is its rigorous methodology, including adherence to PRISMA guidelines and the inclusion of a meta-analytic component. However, limitations such as reliance on English-language studies, retrospective designs, and a lack of studies from low-resource settings (particularly Africa) restrict generalizability. These gaps highlight significant disparities in AI adoption and the urgent need for inclusive datasets and equitable technological development. Future research should prioritize the development of interpretable, externally validated models trained on diverse, population-representative datasets. Strategic investments in data infrastructure, interdisciplinary collaboration, and digital capacity-building are critical, especially in underrepresented regions. Moreover, research should assess the real-world clinical impact, cost-effectiveness, and patient-centered outcomes of AI-assisted RE care. Taken together, AI holds transformative potential in RE management, and with responsible development and global inclusivity, it can serve as a scalable solution to address vision care challenges across diverse healthcare settings."**

Reviewer #2: This paper presents an interesting systematic review of artificial intelligence applications in th diagnosis and management of vision problems associated with refractive errors. The paper is a straightforward review with appropriate methods, a clear PRISMA diagram ,and a readable and informative presentation of results. Analyses by AI techniques, input modalities, and other subgroups provide additional insight, along with descriptive discussions of AI in treatment, and progression of refractive error.

The paper is interesting and informative. I have no concerns with the analysis, or conclusions, but there are a few concerns with some of the presentation, and some additional areas that would merit from further elaboration:

**Thank you very much for the positive remarks on our manuscript and your expert rigorous appraisal which has helped us to extensively improved upon our manuscript.**

1. The figures are of poor quality and should be recreated, or at the very least re-submitted at better resolution. Figures 1 and 2 are pixelated. Figures 3 and 4 and corresponding figures in the supplemental document have a strange aspect ratio, as if they were scaled unevenly in the vertical and horizontal. the SROC curves are hard to read, consisting of what look to be a couple of small squiggles.

**We have recreated Figures 1-4 at high resolution and adjusted the aspect ratios to ensure visual clarity and consistency. We also regenerated the SROC curves with appropriate scale and clearer labeling to improve interpretability.**

2. The early presentation of the results (pages 15-18) is a bit confusing to read, as sections are not as clearly delineated into subsections as might be useful for making the paper more readable.

**Pages 14-20: We have provided correct headings for the subsections.**

3. There are two figures labeled "Figure 4".

**We have correctly numbered the figures.**

4. Tables 2 and 3 provide a useful summary of the papers discussed. However, potentially pertinent details are not included. These tables would be more informative if expanded to include a description of the sample sizes (number of patients/images), and the number of features included.

**Thank you very much for pointing this out. However, the sample sizes (number of patients/images) have been indicated for all included studies in Table 1. We have added the *"number of features"* in the "*input features*" column in Tables 2 and 3.**

5. The paper ends with an argument for the application of AI in African settings. This is certainly an admirable goal worthy of additional discussion. Additional elaboration regarding the factors behind the limited discussion of AI in these settings would be helpful. Specifically, are the limitations associated with a lack of data? A shortage of skilled data scientists capable of creating the appropriate AI models?

**We have expanded the section discussing Africa-specific barriers.**

***We have elaborated on this query in the discussion as seen on pages 31-32, Lines 893-920 and 927 and 932.***

**"Of note, despite the comprehensiveness of our search strategy, we found no evidence of AI utilization in RE management across the African continent. This is particularly concerning given that Africa bears a substantial burden of UREs [102], and faces severe shortages of eye care professionals alongside significant challenges in delivering eye care services [103]. The marked scarcity of AI applications in RE management in this region highlights a significant healthcare disparity that urgently requires attention. In particular, this disparity can be attributed to multiple intersecting challenges, most notably the lack of high-quality, large-scale, and demographically diverse datasets from African populations. Most AI models in RE detection have been developed using datasets from high-income countries, often lacking representation of African ethnic and genetic diversity. As a result, such models may perform poorly when applied in African contexts, potentially leading to biased or inaccurate predictions [104-106]. Furthermore, clinical data collection systems in many African countries are either non-digitized or poorly maintained, with inadequate annotation, storage, and interoperability, limiting their usability for AI model training [104, 107]. In addition to data limitations, there is a notable shortage of trained professionals in AI, data science, and medical image analysis across the continent [106, 108]. Most healthcare workers and researchers have limited exposure to AI model development,**

**validation, or implementation. Moreover, the few individuals who do receive AI training often face challenges such as inadequate infrastructure—limited access to GPUs, cloud computing services, and imaging technologies—further hindering innovation and local model development [107]. Beyond technical limitations, systemic barriers further complicate AI integration in African RE care. These include inadequate policy frameworks, limited funding for digital health innovation, ethical and privacy concerns, and low levels of community trust in AI technologies [109]. Language, cultural relevance, and the usability of AI tools also remain largely unexplored in current implementations, which are often designed for Western users and contexts. The adoption of advanced AI-based RE management systems could help bridge this gap by promoting equitable access to eye care services across Africa, thereby supporting the broader objectives of Sustainable Development Goal (SDG) 3 and the African Union's Agenda 2063 vision for transformed healthcare systems. However, successful implementation of AI in African settings will likely require careful and context-specific planning, taking into account factors such as specialist availability, long-term patient outcomes, and the cost-effectiveness of integrating AI into existing healthcare infrastructures, especially compared to resource-rich countries. Specifically, a multi-faceted strategy is needed. Investments should focus on establishing robust data infrastructure, building AI capacity through education and cross-disciplinary training, and creating inclusive datasets that reflect the diversity of African populations. Partnerships between local institutions, international collaborators, and technology developers can facilitate sustainable and ethical AI integration. Such initiatives will be critical to ensuring that AI tools are not only technically sound but are also socially acceptable and clinically relevant in this region."**

[NOTE: If reviewer comments were submitted as an attachment file, they will be attached to this email and accessible via the submission site. Please log into your account, locate the manuscript record, and check for the action link "View Attachments". If this link does not appear, there are no attachment files.]

Figure resubmission:

While revising your submission, please upload your figure files to the Preflight Analysis and Conversion Engine (PACE) digital diagnostic tool, https://pacev2.apexcovantage.com/. PACE helps ensure that figures meet PLOS requirements. To use PACE, you must first register as a user. Registration is free. Then, login and navigate to the UPLOAD tab, where you will find detailed instructions on how to use the tool. If you encounter any issues or have any questions when using PACE, please email PLOS at figures@plos.org. Please note that Supporting Information files do not need this step. If there are other versions of figure files still present in your submission file inventory at resubmission, please replace them with the PACE-processed versions.

**We have utilized these resources to improve the clarity of our images to meet PLOS Digital Health publishing standards.**

Reproducibility:

To enhance the reproducibility of your results, we recommend that authors of applicable studies deposit laboratory protocols in protocols.io, where a protocol can be assigned its own identifier (DOI) such that it can be cited independently in the future. Additionally, PLOS ONE offers an option to publish peer-reviewed clinical study protocols. Read more information on sharing protocols at https://plos.org/protocols?utm_medium=editorial-email&utm_source=authorletters&utm_campaign=protocols

**We have deposited our study protocol in PROSPERO (CRD 42024592160) for accessibility.**
